# Phosphate availability conditions caspofungin tolerance, capsule attachment and titan cell formation in *Cryptococcus neoformans*

**DOI:** 10.3389/ffunb.2024.1447588

**Published:** 2024-08-14

**Authors:** Xianya Qu, Kabir Bhalla, Linda C. Horianopoulos, Guanggan Hu, Armando Alcázar Magaña, Leonard J. Foster, Leandro Buffoni Roque da Silva, Matthias Kretschmer, James W. Kronstad

**Affiliations:** ^1^ The Michael Smith Laboratories, University of British Columbia, Vancouver, BC, Canada; ^2^ Department of Microbiology and Immunology, University of British Columbia, Vancouver, BC, Canada; ^3^ Department of Biochemistry and Molecular Biology, Metabolomics Core Facility, Life Sciences Institute, University of British Columbia, Vancouver, BC, Canada; ^4^ Department of Biochemistry and Molecular Biology, University of British Columbia, Vancouver, BC, Canada

**Keywords:** pathogenesis, phosphate homeostasis, phospholipid biosynthesis, endoplasmic reticulum (ER) stress, calcineurin, titan cells

## Abstract

There is an urgent need for new antifungal drugs to treat invasive fungal diseases. Unfortunately, the echinocandin drugs that are fungicidal against other important fungal pathogens are ineffective against *Cryptococcus neoformans*, the causative agent of life-threatening meningoencephalitis in immunocompromised people. Contributing mechanisms for echinocandin tolerance are emerging with connections to calcineurin signaling, the cell wall, and membrane composition. In this context, we discovered that a defect in phosphate uptake impairs the tolerance of *C. neoformans* to the echinocandin caspofungin. Our previous analysis of mutants lacking three high affinity phosphate transporters revealed reduced elaboration of the polysaccharide capsule and attenuated virulence in mice. We investigated the underlying mechanisms and found that loss of the transporters and altered phosphate availability influences the cell wall and membrane composition. These changes contribute to the shedding of capsule polysaccharide thus explaining the reduced size of capsules on mutants lacking the phosphate transporters. We also found an influence of the calcineurin pathway including calcium sensitivity and an involvement of the endoplasmic reticulum in the response to phosphate limitation. Furthermore, we identified membrane and lipid composition changes consistent with the role of phosphate in phospholipid biosynthesis and with previous studies implicating membrane integrity in caspofungin tolerance. Finally, we discovered a contribution of phosphate to titan cell formation, a cell type that displays modified cell wall and capsule composition. Overall, our analysis reinforces the importance of phosphate as a regulator of cell wall and membrane composition with implications for capsule attachment and antifungal drug susceptibility.

## 1 Introduction

The opportunistic pathogenic fungus *Cryptococcus neoformans* causes meningoencephalitis in immunocompromised people including those living with HIV/acquired immunodeficiency syndrome (AIDS) or receiving immunosuppressive therapy (Crabtree et al., 2012; Rajasingham et al., 2017; Srikanta et al., 2014; Hommel et al., 2018). In fact, cryptococcal meningoencephalitis accounts for ~19% of the deaths among people living with HIV/AIDS (Srikanta et al., 2014; Esher et al., 2018; Hurtado et al., 2019; Rajasingham et al., 2022). Unfortunately, there are relatively few antifungal drugs to treat cryptococcal disease, and new therapeutic options are needed (Iyer et al., 2021). In this regard, we are interested in identifying the key nutrient acquisition and sensing mechanisms that underpin the pathogenesis of *C. neoformans* and that may provide targets for intervention.

Previously, we examined phosphate acquisition in *C. neoformans* and found that mutants lacking three phosphate transporters were defective in virulence factor production in culture, survival in macrophages, and virulence in a mouse model of cryptococcosis (Kretschmer et al., 2014). Our analysis also revealed connections between phosphate acquisition and regulatory pathways including the cyclic AMP (cAMP)-dependent protein kinase A (PKA), and the calcium-calmodulin-activated protein phosphatase calcineurin. We focused on phosphate because of its metabolic importance, its role in signaling, and because uptake functions are highly expressed during cryptococcosis (Steen et al., 2003; Hu et al., 2008; Yu et al., 2021). Additionally, the regulation of genes in the PHO regulon is well characterized in *Saccharomyces cerevisiae* and other fungi (Secco et al., 2012; Austin and Mayer, 2020). There is considerable overlap in the components of the core PHO regulon in *C. neoformans* and other fungi compared to *S. cerevisiae*, including the key regulator Pho4. This protein interacts with the CDK complex (Pho80-Pho85-Pho81) and regulates the transcription of the genes for the high affinity phosphate transporters Pho84, Pho840, and Pho89, the Vtc1 and Vtc4 proteins involved in polyP (polyphosphate) synthesis, the secreted acid phosphatase Aph1, and the Git1 and Gde1 proteins necessary for phospholipid catabolism (Toh-E et al., 2015; Ikeh et al., 2017; Lev et al., 2017; Lev and Djordjevic, 2018; Bhalla et al., 2022).

A *C. neoformans* mutant lacking all three high affinity phosphate transporters (Pho84, Pho840, and Pho89) demonstrated reduced elaboration of key virulence factors, including the polysaccharide capsule (Kretschmer et al., 2014). Capsule polysaccharides are synthesized intracellularly and trafficked to the cell surface (Yoneda and Doering, 2006; Ramos et al., 2017). Trafficking between the ER and the Golgi apparatus is important for capsule formation as demonstrated by reduced capsule size upon treatment with the inhibitor brefeldin A (Hu et al., 2007). Connections with phosphate availability are evident from the fact that nucleotide sugars such as GDP-mannose, UDP-galactose and UDP-xylose are required for polysaccharide synthesis, and nucleotide sugar transporters (NSTs) deliver NDP-sugars for synthesis, with the generation of free phosphate (Li et al., 2017; Wang et al., 2018). Phosphate acquisition and sensing also contribute to other virulence attributes for *C. neoformans*. For example, phosphate induces the formation of a population of small cells which facilitate dissemination to the brain (Denham et al., 2022). Additionally, a *pho4* deletion mutant shows a smaller cell diameter in infected lung tissues (Lev et al., 2017). Along with the small cells, the fungus also establishes a population of large (titan) cells (> 10 μm in diameter) that contribute to pathogenesis (Okagaki et al., 2010; Zaragoza et al., 2010; Crabtree et al., 2012; Okagaki and Nielsen, 2012; Dambuza et al., 2018; Hommel et al., 2018; Dyląg et al., 2020).

In this study, we examined the impact of deletion of the three phosphate transporters on capsule formation in more detail and found that phosphate reduced polysaccharide attachment to the cell wall resulting in increased shedding in culture supernatant. An impact on the cell wall was evident from the decreased tolerance of the transporter mutants to the antifungal drug caspofungin. This drug inhibits β-1,3-glucan synthesis, and *C. neoformans* is tolerant to the drug (Del Poeta et al., 2000; Kartsonis et al., 2003; Formosa et al., 2013; Walker and Munro, 2020; Kalem et al., 2021; Papon and Goldman, 2021; Mukaremera, 2023). We further characterized connections between capsule formation, endoplasmic reticulum (ER) stress (including the Unfolded Protein Response (UPR)) and phosphate homeostasis. Specially, we found that tunicamycin, an inhibitor of N-linked glycan biosynthesis in the ER, reduced capsule formation and shed polysaccharide. Observed changes in plasma membrane integrity, lipid composition, and cell wall structure in different phosphate conditions were also consistent with altered phosphate homeostasis in the mutants lacking phosphate transporters. Furthermore, we found connections between calcium-calcineurin signaling and the PHO pathway as established in yeast and identified previously in *C. neoformans* (Serrano et al., 2002; Huang et al., 2007; Kretschmer et al., 2014; Bowring et al., 2023). Finally, our results also revealed that phosphate availability influenced titan cell formation. Overall, these studies establish connections between phosphate availability, cell wall and membrane composition, and ER stress that influence the key virulence properties of capsule and titan cell formation.

## 2 Materials and methods

### 2.1 Strains and growth medium

Strain H99 of *C. neoformans* var. *grubii* (serotype A) and derived mutants were used in the experiments. Previous gene deletion mutants lacking high affinity phosphate transporters (*pho84*Δ*, pho840*Δ*, pho89*Δ*, pho84*Δ*840*Δ*, pho84*Δ*89*Δ and *pho84*Δ*840*Δ*89*Δ *(pho*ΔΔΔ*))* were used in this study (Kretschmer et al., 2014). Strains were grown in yeast extract-peptone-dextrose (YPD) (1% yeast extract, 2% peptone, 2% dextrose, 2% agar, broth or agar, pH 7.0) or yeast nitrogen base (YNB, Difco) with/without amino acids (broth or agar, pH 5.6). Minimal medium (MM, 15mM D-glucose, 10 mM MgSO_4_, 29.4mM KH_2_PO_4_, 13mM glycine, 3.0 μM thiamine, pH 5.5) was used to induce titan cell formation . Capsule was induced using defined low-iron capsule-inducing medium (LIM) (0.5% glucose, 38 mM L-asparagine, 2.3 mM K_2_HPO_4_, 1.7 mM CaCl_2_·2H_2_O, 0.3 mM MgSO_4_·7H_2_O, 20 mM HEPES, 22 mM NaHCO_3_, 1 ml of 1000X salt solution (0.005 g/L CuSO_4_·5H_2_O, 2 g/L ZnSO_4_·7H_2_O, 0.01 g/L MnCl_3_·4H_2_O, 0.46 g/L sodium molybdate, 0.057 g/L boric acid), in iron-chelated dH_2_O adjusted to pH 7.4 (with 0.4 mg/L sterile thiamine added) prepared as previously described (Lian et al., 2005). Phosphate levels were provided and adjusted with KH_2_PO_4_ and Pi indicates inorganic phosphate. Dulbecco’s Modified Eagle Medium (DMEM, Gibco) supplemented with 10% Fetal Bovine Serum, 100 units/ml penicillin, 100 μg/ml streptomycin, and 2 mM L-glutamine at 37°C with 5% CO_2_ was used for propagation of the macrophage-like cell line J774A.1 (ATCC, Manassas, Virginia). All chemicals were from Sigma-Aldrich (St. Louis, MO, USA) unless otherwise stated.

### 2.2 Assessment of capsule formation

Capsule formation was examined by differential interference contrast microscopy (DIC). Cells were grown in YPD at 30°C overnight, washed three times with LIM, and transferred into 2 ml LIM. After incubation, cells were imaged with India ink staining using a Zeiss Plan-Apochromat 100×/1.46 oil lens on a Zeiss Axioplan 2 microscope.

### 2.3 Assessment of capsule shedding

Cells were grown in YPD overnight, washed three times with LIM, transferred to 3 ml LIM, and incubated at 30°C for 48 h. Supernatant from each culture (normalized to an OD_600_ of 2) was denatured at 70°C for 15 min, subjected to electrophoresis on a 1% agarose gel, and blotted onto a nylon membrane (GE Healthcare, Boston, MA, USA). The membrane was incubated with a 1:5,000 dilution of the 18B7 monoclonal antibody, followed by incubation with a 1:5,000 dilution of anti-mouse HRP (Bio-Rad). Bound polysaccharide was visualized by chemiluminescence (GE Healthcare, Boston, MA, USA).

### 2.4 Orthophosphate measurements

Orthophosphate levels were measured by molybdate reactivity (Reddi et al., 2008; Rosenfeld et al., 2010). Cells were grown in YPD overnight and transferred into YNB with different phosphate levels for 24 h. Cells (OD_600_ of 4) were harvested, washed twice with dH_2_O and resuspended in 500 μL of 0.1% Triton X-100. Cells were lysed by glass bead homogenization and lysates were clarified by centrifugation for 5 minutes at 15,000 rpm. The samples were normalized with to the same protein amount (100 µg) using the Pierce™ BCA Protein Assay Kit. A buffer solution (1 mL) of 0.1 M NaCl, 0.5 mL 0.5 M H_2_SO_4_, 0.5 mL 50 mM (NH_4_)_6_Mo_7_O_24_-4H_2_O, and 3 mL 1-butanol was added to each sample. Then, 1 mL of the upper phase was collected and added to 0.5 mL of 9 mM SnCl_2_-H_2_O. After vortexing, the absorbance of the upper phase was measured at 700 nm and the inorganic phosphate levels were calculated based on standard curves generated from phosphoric acid standards.

### 2.5 Transmission electron microscopy

Cells were grown overnight in YPD, transferred to MM with 0 mM phosphate (Pi) or 250 mM Pi provided as KH_2_PO_4_ at 30°C and normalized to an OD_600_ of 2, using previously reported concentrations and buffers (Kretschmer et al., 2014). After 24 h, cells were washed three times in sterile dH_2_O and fixed in 2.5% glutaraldehyde in 0.1 M sodium cacodylate pH 6.9 for 1 h at room temperature (RT). The fixative solution was removed by 3 washes with 0.1M sodium cacodylate buffer. After fixation, cells were separated in 3% low temperature gelling agarose and post fixed with 1% OsO_4_, 0.8% K_4_[Fe (CN)_6_], 5mM CaCl_2_ in 0.1 M sodium cacodylate at pH 7.4 for 10 minutes. The cells were washed three times with dH_2_O and incubated in 1% thiocarbohydrazide (TCH) in dH_2_O for 5 minutes and then washed 3x in dH_2_O and then post-fixed again for 2 minutes. After 3 washes with dH_2_O, the cells were dehydrated through sequential washes with a graded concentration series of acetone into 100% ethanol. After dehydration, cells were embedded in Spurr’s resin and 70 nm sections were cut using a Leica Ultramicrotome UCT. Sections were stained with 2% uranyl acetate (UA) for 12 minutes, followed by Reynolds’ Lead Citrate for 6 minutes. Images were taken on a FEI Tecnai Spirit 120kV Transmission Electron Microscope operated at an accelerating voltage of 80kV and images were acquired with a DVC1500M side-mounted camera controlled by AMT software.

### 2.6 RNA extraction and RT-qPCR

Cells were grown in YPD media overnight until log phase, washed twice in YNB with 20 mM Pi, and transferred to YNB with 20 mM Pi and 2% glucose. After 24 h of starvation at 30°C with shaking, 2х10^7^ cells were transferred to YNB with 20 mM Pi (low phosphate) or 250 mM Pi (high phosphate) at 30°C for Pi treatment. Cells were harvested, frozen in liquid nitrogen, and stored at −80°C. RNA was extracted at 0.5 h, 2 h and 5 h with lysis buffer (2% SDS, 68 mM tri-sodium citrate, 132 mM citric acid, 10 mM EDTA, pH 3.5) and extraction buffer (4 M sodium chloride, 17 mM tri-sodium citrate, 33 mM citric acid, pH 3.5). DNase treatment was performed with Turbo DNase (Ambion, Austin, TX) according to the manufacturer’s instructions, and cDNA synthesis was performed using the Verso cDNA reverse transcription kit (Thermo Fisher Scientific, Waltham, MA) with oligo (dT). Quantitative reverse transcription PCR (qPCR) was performed using the Green-2-Go qPCR Mastermix with the primers listed in the **Supplementary Table S1**. Glyceraldehyde phosphate dehydrogenase (GAPDH) and actin (ACT1) were used for normalization. The following genes were analyzed (*PHO84, PHO89, PHO840, PHO81*, *PHO91*, *CIR, PKA1, PKR, RIM101, VTC4, CAP60, CAP64, CAS35, IRE1, HXL1_1* and *HXL1_2*) and relative transcript levels were quantified using the 2^−ΔΔCT^ method.

### 2.7 Drug and metal assays

YPD agar was used with the following concentrations of reagents for testing sensitivity: 125 ng/ml tunicamycin; 15 mM DTT (ER stress); SDS (0.01%, 0.005% and 0.0001%, plasma membrane stress); 1.5 M KCl, 1.5 M NaCl, 1 M, 1.5 M sorbitol (osmotic stress); 1 mM NaNO_2_, 4 mM NaNO_2_ (nitrosative stress, pH 4); 2 mM H_2_O_2_ (oxidative stress); 0.5% Congo Red, 1 mg/ml calcofluor white (CFW), 0.5 mg/ml caffeine (cell wall stress); 5 mM MnCl_2_, 5 mM ZnCl_2_, 5 mM NiSO_4_ (metal stress); 100 nM rapamycin (inhibitor of mTOR); CaCl_2_ (50 mM, 100 mM, 200 mM, 400 mM); calcium chelator EGTA [5 mM, ethylene glycol-bis (β-aminoethyl ether)-N, N, N′,N′-tetraacetic acid]; 1 µg/ml tacrolimus (FK506), 100 µg/ml of cyclosporin A (CsA), 10 μg/ml fluconazole, and 0.5 μg/ml amphotericin B (antifungal drugs). Briefly, cells were grown in YPD overnight, washed three times with sterile dH_2_O, counted and adjusted to 2х10^7^ cells/ml in dH_2_O. Serial dilutions (1:10) were performed and 5 µl (3 µl for SDS plates) of each dilution was spotted onto agar medium (10^5^ cells for first spot). Plates were incubated at 30°C, 37°C or 39°C for 3 days.

### 2.8 Growth assays

ER stress was tested with 125 ng/ml tunicamycin in YPD media. Other drugs such as nikkomycin, manumycin and foscarnet were tested in 96 well plates with YNB medium containing 2% glucose and 100 mM Pi provided as KH_2_PO_4._ A 1:2 serial dilution was performed on these three drugs with starting concentrations of 200 µg/ml, 3 µg/ml and 500 µg/ml, respectively. For growth assays in test tubes, cells were grown in YPD overnight, washed three times with dH_2_O, counted and adjusted to 10^6^ cells. Cells were placed into 3 ml of the corresponding media, and incubated at 30°C and 37°C for up to 96 h. In 96 well plates, cells were grown in YPD overnight, washed three times with dH_2_O, counted and adjusted to 2х10^7^ cells/ml. 5 μl of cells were added into each well with a corresponding media volume of 200 μl (10^5^ cells per well) with drugs as indicated. 96 well plates were incubated at 30°C for up to 96 hours and the OD_600_ values were recorded every 24 h using a microplate reader (Infinite M200, Tecan).

### 2.9 Flow cytometry and microscopy

Cells were grown overnight in YPD and transferred to various media (1 x 10^7^ cells in 500 μl) for staining with the procedures and dyes listed in **Supplementary Table S2**. Aniline blue (0.05%) staining was used to measure -1,3-glucan, as previously described (O’Meara et al., 2013). Samples were washed 3x with PBS post staining and analyzed on an Attune Nxt Flow Cytometer (Invitrogen, Carlsbad, CA) using a PB450 filter. Chitin and chitosan levels were determined by flow cytometry with calcofluor white (CFW) (PB450, 405/450 nm) and eosin Y (FITC, 488/530 nm) staining, respectively. Cells were pre-grown in YPD and then transferred into minimal media (YNB, with different phosphate levels and buffered with 50 mM MOPS to pH 7.0) for 24 h.

### 2.10 Cell preparation for liquid chromatography-high resolution and mass spectrometry

The lipidomics analysis was performed with cells grown overnight in YPD, transferred to MM at 30°C, and normalized to an OD_600_ of 2 with 0 mM or 250 mM Pi. After 24 h of incubation, 2 ml of cells were transferred (normalized to an OD_600_ of 2) into a 2 mL microcentrifuge tube. For metabolomics, cells were grown overnight in YPD, transferred into LIM (with 2.5 mM Pi) with iron (using dH_2_O instead of low iron water) for 24 h. 5х10^7^ cells were transferred into LIM (low iron, low Pi), LIM (low iron, 20 mM Pi), LIM (iron, low Pi) and LIM (iron, 20 mM Pi) for 6h. 3.5х10^7^ cells were collected into a 2 mL microcentrifuge tube. Cells were centrifuged at 13,000 rpm, 4°C for 10 min, washed three times with ice-cold nanopure water. Lipid extraction was performed using a biphasic system of cold methanol, methyl tert-butyl ether (MTBE), and H_2_O, as described with some modifications (Matyash et al., 2008). In brief, 300 μl ice cold methanol containing 0.05 mg/mL of BHT was added to the cell pellet and vortex at 4°C for 2 minutes, followed by addition of 150 µl glass beads. Cells were homogenized using a bead beater for 20 seconds three times with 30 s on ice between cycles. Then, 600 μL ice cold MTBE was added, vortexed and shaking at 4°C for 10 min on an orbital shaker, followed by the addition of 200 μl ice cold nanopure water to allow the formation of the two phases followed by centrifugation at 14,000 rpm for 10 min at 4°C to remove beads and cell debris. The organic phase (upper layer) was collected for lipidomics, and lower layer was collected for metabolomics; each phase was dried in a speedvac at RT. Samples were stored at -80°C. Prior to LC−MS/MS analysis, lipid extracts were resuspended using a mixture of acetonitrile:isopropanol (7:3, v/v) containing 200 ng/mL of CUDA (12-[[(cyclohexylamino)-carbonyl]amino]-dodecanoic acid) as an internal standard. The samples were vortexed for 10 min and centrifuged at 14,000 rpm for 10 min. Aliquots of 50 µL from each sample were pooled to generate a quality control sample (QC) used for evaluating the LC–MS/MS platform performance.

### 2.11 Liquid chromatography and high-resolution mass spectrometry for lipidomics analysis

Untargeted lipidomics analysis was performed using an IMPACII™ high-resolution spectrometer from Bruker Daltonics (Bruker Daltonics, Bremen, Germany) coupled with an Elute UHPLC system (Bruker Daltonics). Separation of compounds was achieved using a multigradient method on an Acquity CSH (charged surface hybrid) C18 column (130Å, 1.7 µm, 100 x 2.1 mm) (Waters, Milford, MA) equipped with a CSH C18 VanGuard FIT Cartridge (1.7 µm, 2.1 x 5 mm). With some modifications, analysis was performed according to Cajka et al (Cajka et al., 2017). The mobile phase A consisted of acetonitrile: water 60:40 (v/v). Phase mobile B was isopropanol: acetonitrile 90:10 (v/v), both phases containing 0.1% v/v formic acid and 10 mM ammonium formate. The separation was conducted using a gradient ranging from 15% to 99% mobile phase B over 17 minutes as follows: 0 min 15% B; 0–2 min 30% B; 2–2.5 min 50% B; 2.5–12 min 80% B; 12–12.5 min 99% B; 12.5–13.5 min 99% B; 13.5–13.7 min 15% B; 13.7-17 min 15% B. The column temperature was set to 65°C, and the flow rate was 0.5 mL min^-1^. The injection volume was 2 µL, while the autosampler was maintained at 4°C.

To evaluate instrument performance, aliquots of 50 µL from each sample were pooled to create a quality control sample (QC). This sample was injected every six samples. Data-dependent acquisitions were conducted in positive (ESI+) and negative (ESI-) ionization modes to obtain precursor and fragment ion information for annotating compounds. For ESI+, the following mass spectrometer settings were used: capillary voltage of 4500 V, nebulizer gas pressure of 2.0 bar, dry gas flow rate of 9 L min^-1^, dry gas temperature of 220°C, mass scan range of 65-1700 *m/z*, spectra acquisition rate of of 3 Hz, and cycle time of 0.7 s. Collision energy of 20 V was ramped through each MS/MS scan from 100 to 250%. For ESI-, the capillary voltage was set at -4000 V. Calibration was performed by injecting 10 µL of 10 mM sodium formate at the beginning of each run via the 6-port diverter valve.

### 2.12 Liquid chromatography-high resolution mass spectrometry for nucleotide sugar and metabolite analysis

A novel method optimized for highly polar compounds was used to analyze samples using Hydrophilic Interaction Liquid Chromatography (HILIC). A mixture of commercially available standards of sugar nucleotides at 1 ppm was administered with a syringe pump to establish optimal performance allowing fine-tuning of the method and low detection limits. Samples were analyzed using an IMPACII™ high-resolution mass spectrometer (Bruker Daltonics, Bremen, Germany), coupled with a Vanquish UHPLC system (Thermo Fisher Scientific, Waltham, MA). Separation of compounds was accomplished with an InfinityLab Poroshell 120 HILIC-Z column (2.7 µm particle size, 150 x 2.1 mm) from Agilent. The mobile phase consisted of two components: (A) water with 0.1% v/v formic acid, 10 mM ammonium acetate, and 5 µM medronic acid, and (B) 90% acetonitrile with 10% water v/v supplemented with 0.1% v/v formic acid, 10 mM ammonium acetate, and 5 µM medronic acid. A multi-step gradient was used for separation: the initial condition of 90% B was held for 2 minutes, followed by a linear gradient to 40% B over 6 minutes, then maintained at 40% B for 2 minutes, returned to 90% B over 1.1 minutes, and equilibrated for 4.9 minutes, resulting in a total run time of 16 minutes. The column temperature was maintained at 30°C, the autosampler at 4°C, and the flow rate was set at 0.3 ml min^-1^.

Data-dependent acquisitions were conducted in both negative (ESI-) and positive (ESI+) ionization modes to obtain precursor and fragment ion information for compound annotation. Sugar nucleotides were measured in negative ion mode. For ESI-, the mass spectrometer settings were: capillary voltage of -3800 V, nebulizer gas pressure of 2.0 bar, dry gas flow rate of 9 L min^-1^, dry gas temperature of 220°C, mass scan range of 100-1500 *m/z*, spectra acquisition rate of 3 Hz, and cycle time of 0.7 s. Collision energy was ramped from 20 V through each MS/MS scan from 100 to 250%. Quadruple ion energy was set at 4 eV with a pre-pulse storage time of 7 µs. Collision RF and transfer time were ramped from 250 Vpp and 40 µs (15% of the time) to 850 Vpp and 115 µs (85% of the time), respectively. For ESI+, the capillary voltage was set at 4500 V. Internal calibration was performed in every sample by injecting 10 µL of 10 mM sodium formate at the beginning of each run via a 6-port diverter valve.

### 2.13 Metabolite identification and data processing

Raw data processing of metabolites employed Progenesis QI™ (V3.0.7600.27622) software with the METLIN™ plugin (V1.0.7642.33805). This involved deconvolution, peak picking, alignment, normalization, and database searching as previously described (Magana et al., 2020; Abbaoui et al., 2018). Annotations were carried out using experimental MS/MS data for querying and matching against METLIN™ (Montenegro-Burke et al., 2020), Lipidblast (Kind et al., 2013), MS-DIAL (Tsugawa et al., 2015), GNPS (Wang et al., 2016), HMDB (Wishart et al., 2022) and MassBank of North America [https://mona.fiehnlab.ucdavis.edu/]. To increase the confidence on the annotations, molecular features without MS/MS were excluded from downstream analysis. Annotations with a Progenesis QI score of 40 or more were considered for selection as level 2 annotations, in accordance with the Metabolomics Standards Initiative (MSI) reporting criteria (Wishart et al., 2022). Annotations for which identity was confirmed with authentic standards were considered as level 1 including the nucleotide sugars. Only ions generated from QC samples with a coefficient of variation (CV) for abundance of less than 25% were included in the dataset. In cases where compounds were detected in both ion modes, the one with lower CV was selected. Finally, the relative quantities of metabolites were determined by calculating their corresponding peak areas in Progenesis QI™.

### 2.14 *In vitro* titan cell formation assay

Cells were grown on Sabouraud Dextrose Agar (SDA, 4% dextrose (glucose), 1% peptone, 1.5% agar) for 2-5 days. Then 10^7^ cells were suspended in 10 mL of liquid YPD medium in a T25 cm^3^ flask and incubated under agitation (150 rpm) at 30˚C for 22 h until stationary phase (final concentration of 2x10^8^ cells/mL). Subsequently, 10^6^ cells were resuspended in 1 mL of MM in a 1.5 mL Eppendorf tube and incubated at 800 rpm for 48 h using an Eppendorf thermomixer. Cells were imaged with India ink staining using a Zeiss Plan-Apochromat 100×/1.46 oil lens on a Zeiss Axioplan 2 microscope. Cells with body sizes >10 μm were considered to be titan cells, and cell size was measured and analyzed by ImageJ (n=50).

### 2.15 Macrophage phagocytosis assay

Intracellular proliferation assays were performed using the murine macrophage-like cell line J774A.1 (ATCC, Manassas, Virginia) as previously described (Caza et al., 2016; Hu and Kronstad, 2010; Hu et al., 2021). *Cryptococcus* strains were grown in YPD overnight at 200 rpm 30°C while J774A.1 cells were seeded to 1 x 10^5^ cells/ml per well (three wells for each treatment, 1 ml in each well) in a 24 well plate, incubated at 37°C, 5% CO_2_ for 24 h prior to infection. *Cryptococcus* cells were opsonized by IgG anti-crypto 18B7 with a final concentration of 10 μg/ml while J774A.1 cells were activated with 150 ng/ml of phorbol 12-myristate 13-acetate (PMA; Sigma-Aldrich, St. Louis, MO) in serum free DMEM (sfDMEM), incubated at 37°C, 5% CO_2_ for 1 hour. Macrophages were then incubated with *C. neoformans* strains for 2 h at a multiplicity of infection 1:1 (macrophage:yeast) at 37°C with 5% CO_2_. Immediately after incubation (time 0 h, T0), external yeast cells were washed off with sterile PBS (4-5 washes) and half the macrophages were lysed with sterilized distilled water to collect phagocytosed fungal cells. After 20 h of incubation, sterile, ice‐cold distilled H_2_O was applied to each well to lyse the macrophages (confirmed microscopically). Fungal growth was measured by plating cells on YPD and determining CFUs. For phagocytosis imaging, J774A.1 cells were seeded to 1х10^5^ cells/ml per well (500 μl in each well) in an 8 well chamber (Nunc™ Lab-Tek™ II Chamber Slide™ System, Thermo Fisher Scientific, Waltham, MA), incubated at 37°C, 5% CO_2_ for infection. After 2 h of infection, samples were imaged by ZEISS Blue microscope and analyzed by ImageJ. Statistical significance was determined by performing with Tukey’s multiple comparisons in GraphPad Prism 8.0 (GraphPad Software).

### 2.16 Murine infection model and histopathology

For virulence assays, inocula were prepared by growing WT and *pho*ΔΔΔ*_29b, pho*ΔΔΔ*_44* mutant cells in YPD overnight at 30°C, washing three times in sterile PBS (Gibco, Waltham, MA, United States), and resuspending at 4.0×10^6^ cells/ml in PBS. Three female BALB/c mice aged 4–6 weeks old (Charles River Laboratories, ON, Canada) were anesthetized intraperitoneally with 80 mg/kg ketamine and 5.5 mg/kg xylazine and inoculated with each strain by intranasal instillation with 50 μl of cell suspension (inoculum of 2×10^5^ cells per mouse). Infected mice were monitored daily post-inoculation and were euthanized by carbon dioxide anoxia upon displaying signs of morbidity. For the determination of fungal burdens in organs at the endpoint, the brain, lung, liver, kidney and spleen were aseptically removed, weighed, and immersed in PBS. After homogenized in two volumes of PBS using a MixerMill MM400 (Retsch, Haan, Germany), serial dilutions of the homogenates were plated on YPD agar plates containing 50 μg/ml chloramphenicol. After 2 d of incubation at 30°C, the colony-forming units (CFU) were counted manually. Histopathology was performed with lung tissues. Lungs of infected mice were isolated, fixed, and stained with mucicarmine or hematoxylin and eosin (H&E) to investigate the progression of infection for the WT or the *pho*ΔΔΔ strain at the humane endpoint (>15% weight loss plus fast breathing). All experiments with mice were conducted in accordance with the guidelines of the Canadian Council on Animal Care and approved by the University of British Columbia’s Committee on Animal Care (protocol A21-0105).

### 2.17 Statistics

Image J was used to measure cell diameter, cell wall size, and capsule thickness. All assays including spot assays, capsule measurements, growth curves, flow cytometry, phagocytosis, microscopy, and qPCR were performed three times. Means and standard deviations are shown, and statistical significance was determined by one way ANOVA and Tukey’s multiple comparisons in GraphPad Prism 8.0 (GraphPad Software).

## 3 Results

### 3.1 Defects in phosphate transport influence caspofungin tolerance and cell wall composition

**Figure 1:**
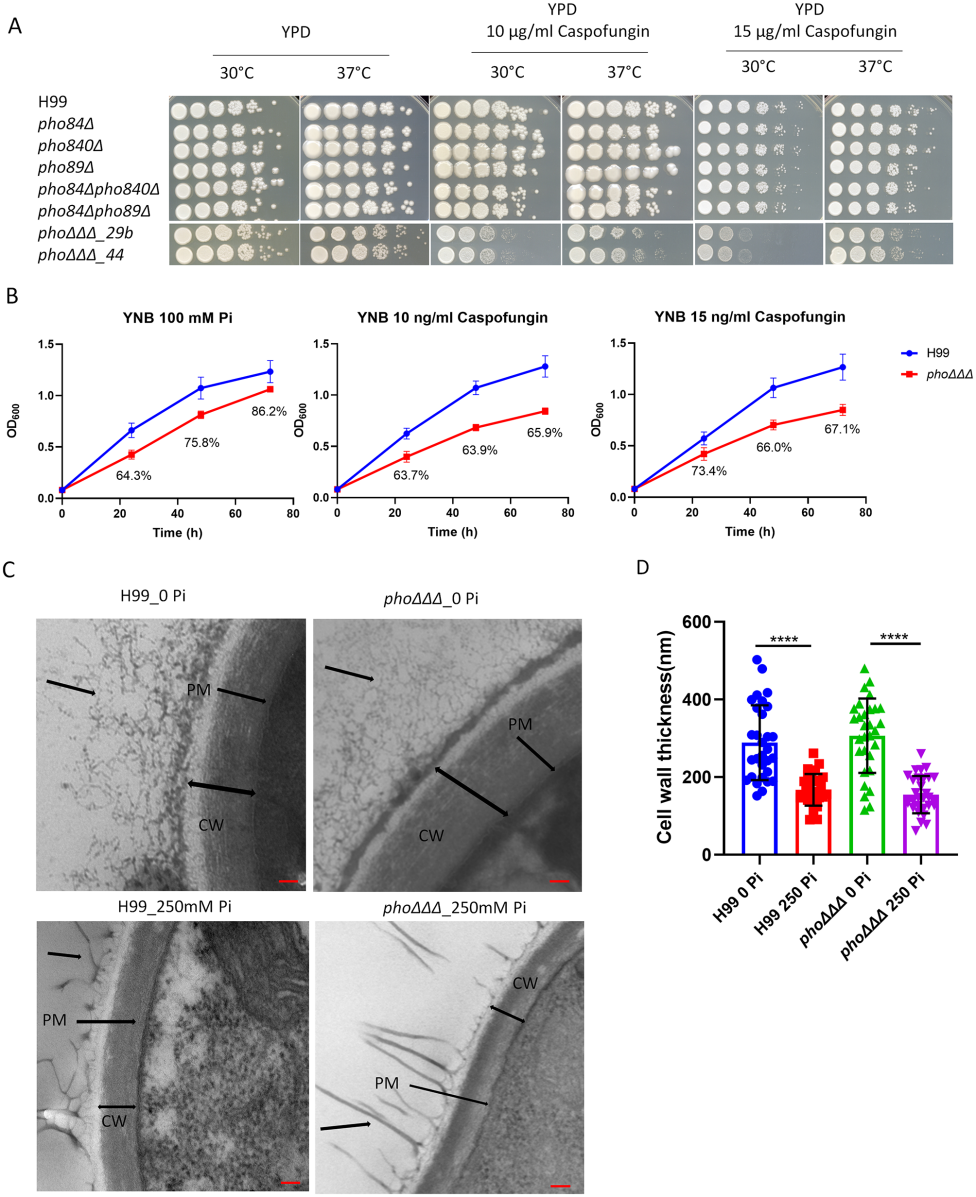
Mutants lacking three high-affinity phosphate transporters are susceptible to caspofungin. **(A)** Assays to test the tolerance of the single, double and triple mutants to the antifungal drug caspofungin. The plates were incubated at 30°C or 37°C for 3 days and then photographed. The strains labeled *pho*ΔΔΔ*_29b* and *pho*ΔΔΔ*_44* are two independent mutants. **(B)** Growth assays in YNB at 30°C (100 mM phosphate) for the *pho*ΔΔΔ mutant in the presence of caspofungin. The culture OD_600_ of the WT (H99) strain and the *pho*ΔΔΔ mutant were measured every 24 (h) The percentages indicate the extent of lower growth for the mutant at each time point. For both **(A)** and **(B)**, the assays were repeated three times and representative results are shown. **(C)** Representative images of the cell walls obtained by transmission electron microscopy (TEM), scale bar = 100 nm (CW = cell wall, PM = plasma membrane, black arrow points at potential capsule structure). **(D)** The cell wall thickness of the WT strain and deletion mutant were measured from **(C)** (n=30). The thickness of the cell wall, mean and standard deviation of measurements for each group are shown. Statistical significance was determined using one-way ANOVA with Tukey’s multiple comparisons in GraphPad Prism 8.0 (GraphPad Software) (****p<0.0001).

We previously characterized mutants lacking the high affinity phosphate transporters (*PHO89, PHO84* and *PHO840)* and found that the triple mutant (designated *pho*ΔΔΔ) displayed several phenotypes related to virulence including reduced capsule and melanin formation (Kretschmer et al., 2014). To better understand the root causes of these virulence factor defects, we tested the sensitivity of the *pho*ΔΔΔ mutant to a more extensive range of drugs and stressors and found that the mutant was sensitive to SDS, NaCl, KCl, sorbitol, NaNO_2_, tunicamycin and fluconazole (**Supplementary Figures S1A-C**). The *pho*ΔΔΔ mutant also showed resistance to rapamycin, zinc, and nickel, but there was no impact of amphotericin B on growth (**Supplementary Figures S1A-C**). Notably, the *pho*ΔΔΔ mutant did not show growth defects on caffeine or calcofluor white (CFW), but did show a color change on congo red (**Supplementary Figure S1A**). The latter phenotype focused our attention on potential changes in cell wall composition and we therefore examined the mutants for sensitivity to the glucan synthase inhibitor caspofungin. Interestingly, the *pho*ΔΔΔ mutant showed decreased tolerance to caspofungin (**Figures 1A, B**), and we noted that the mutant was slightly less sensitive at 37°C, compared with 30°C (**Figure 1A**). Cell wall thickness was also compared by TEM for the WT strain and the mutant after growth in low (0 Pi) and high (250 mM Pi) phosphate conditions (**Figure 1C**). A significant difference was not found between the WT strain and the mutant, but high phosphate induced thinner cell walls in both strains (**Figures 1C, D**).

**Figure 2:**
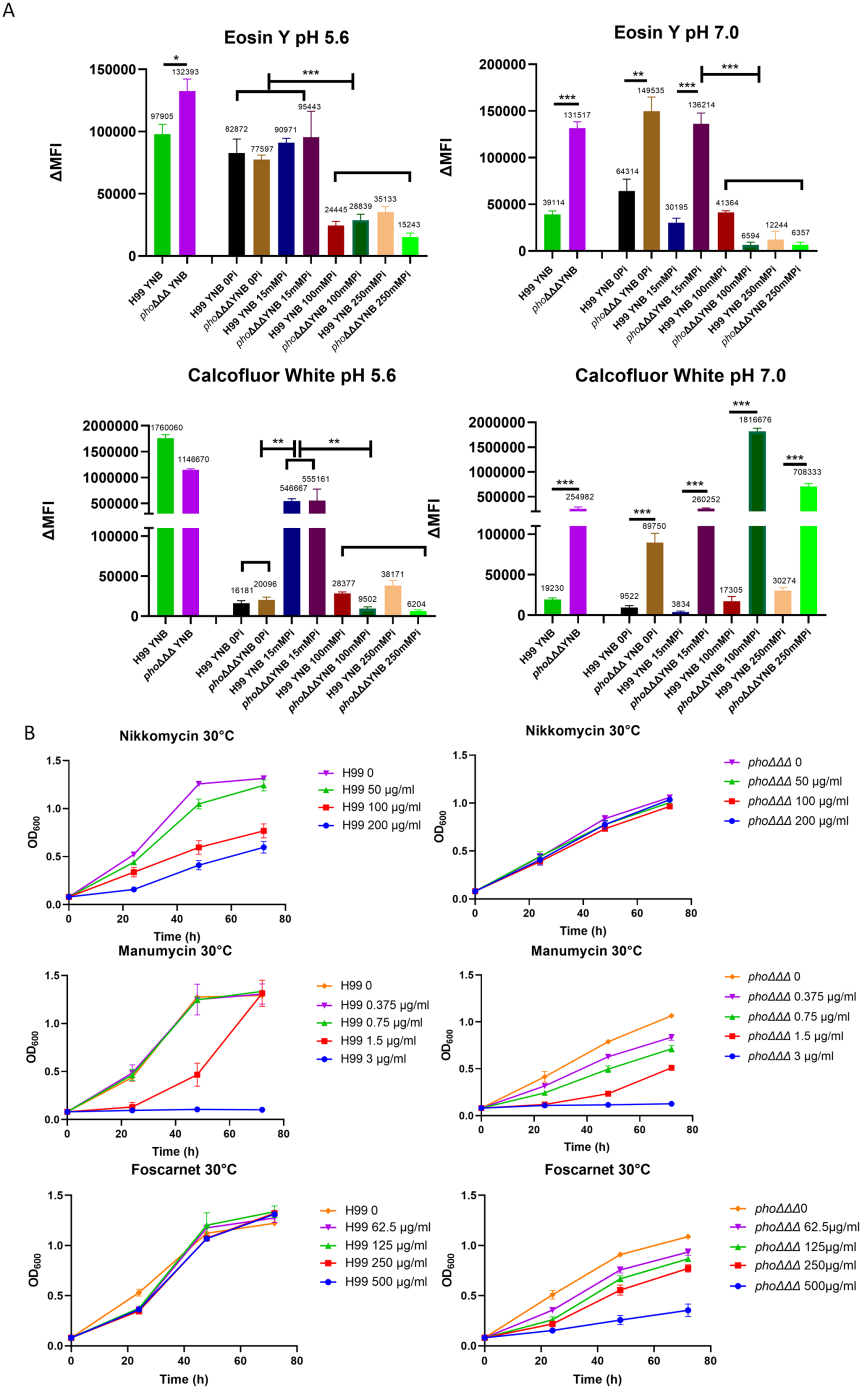
The architecture of the cell wall differs between WT and mutant strains. **(A)** Levels of chitin (CFW) and chitosan (eosin Y) were determined by flow cytometry with Pacific Blue (PB450, 405/450 nm) and fluorescein isothiocyanate (FITC, 488/530 nm), respectively. Cells were pre-grown in YPD and then transferred into minimal media (YNB, with different phosphate levels and two pH conditions) for 24 h and the assays were repeated three times with three biological replicates each. ΔMFI = change in mean fluorescence intensity. Error bars represent standard deviation. **(B)** Growth of the WT strain and the *pho*ΔΔΔ mutant on the indicated concentrations of nikkomycin, manumycin and foscarnet. Cells of the WT strain (H99) and the *pho*ΔΔΔ mutant were grown in YPD overnight, washed and transferred into YNB with 100 mM phosphate in 96 well plates for the growth assays (30°C). The OD_600_ was measured every 24 (h) The assays were repeated three times with three biological replicates each. Statistical significance was determined using one way ANOVA with Tukey’s multiple comparisons in GraphPad Prism 8.0 (GraphPad Software) (ns, non-significant; *p<0.05 **p<0.01 and ***p<0.001).

The sensitivity to caspofungin prompted a closer examination of the cell wall. A recent study indicated that the cell wall in *C. neoformans* is influenced by media composition and pH (Upadhya et al., 2023). Consistent with these findings, we used flow cytometry to demonstrate that the WT strain had less staining with both CFW and eosin Y at pH 7.0, compared with pH 5.6, indicating less chitin and chitosan, respectively (**Figure 2A**). The level of chitin in the *pho*ΔΔΔ mutant showed a similar responsiveness to pH and demonstrated a unique response to high phosphate (100 or 250 mM). That is, the *pho*ΔΔΔ mutant generally displayed higher CFW staining than the WT strain at pH 7.0 (**Figure 2A**). Staining with either CFW or eosin Y was lower in the WT strain at the highest phosphate levels and we noted a similar response for the *pho*ΔΔΔ mutant for eosin Y staining (**Figure 2A**). For comparison, we examined the acapsular mutants *cap59*Δ and *cap60*Δ and found that they did not show differences from WT with eosin Y staining except at the highest phosphate level. However, greater CFW fluorescence intensity was observed for the mutants versus WT in both YPD and YNB media and at all phosphate levels (**Supplementary Figure S2**). The differences may reflect changes in cell wall composition, although it is also possible that the CFW dye might more easily penetrate to the cell surface because the mutants lacked a capsule.

Our experiments generally revealed that the WT cells had less CFW and eosin Y staining upon growth in the high phosphate conditions at pH 5.6. This result is consistent with the conclusion that phosphate availability influences cell wall composition, as also indicated by the TEM observations (**Figure 1C**). In this context, aniline blue staining revealed that the *pho*ΔΔΔ mutant showed greater staining compared to WT, perhaps indicating that β-glucan was more exposed in the mutant due to altered capsule structure (**Supplementary Figure S3**). For both the mutant and WT, the aniline blue fluorescence intensity was higher when phosphate increased (3–15 mM), but decreased when phosphate reached 100 mM, suggesting that phosphate may alter the β-glucan level in the cell wall. As with the staining assays, it is possible that greater drug access in the absence of a capsule may also explain in part why the *pho*ΔΔΔ mutant was more sensitive to caspofungin.

Connections between phosphate, the cell wall, and loss of caspofungin tolerance were further tested with pharmacological agents previously shown to influence the cell wall and/or condition caspofungin sensitivity (Pianalto et al., 2019). The assays were performed in media containing 100 mM phosphate and we found that the chitin synthase inhibitor nikkomycin A impaired growth of the WT strain at both 30°C and 37°C (**Figure 2B**, **Supplementary Figure S2**). In contrast, growth of the *pho*ΔΔΔ mutant was not inhibited, a finding consistent with altered chitin deposition identified in **Figure 2A**. Another agent, manumycin, a protein farnesyltransferase inhibitor which interferes with localization of Ras1 on plasma membrane (Hast et al., 2011), is known to synergize with caspofungin (Pianalto et al., 2019). The *pho*ΔΔΔ mutant was more sensitive than WT to manumycin and this phenotype may indicate an impact of impaired phosphate uptake on the plasma membrane (**Figure 2B**, **Supplementary Figure S2**). We also tested phosphonoformic acid (foscarnet), an antiviral drug that inhibits the Pho84 phosphate transporter in *C. albicans* (Liu et al., 2018), and found increased sensitivity of the *pho*ΔΔΔ mutant compared to WT (**Figure 2B**). This result suggests that additional low affinity transporters may be targeted by foscarnet in *C. neoformans* or that another activity of the drug (e.g., inhibition of pyrophosphate cleavage from dNTPs) may inhibit mutant growth. Taken together, these results support an impact of phosphate availability on cell wall composition, perhaps due to an influence on the availability of nucleotide sugars for wall synthesis, as observed in other fungi (Liu et al., 2020).

### 3.2 Phosphate suppresses capsule size and increases polysaccharide shedding

**Figure 3:**
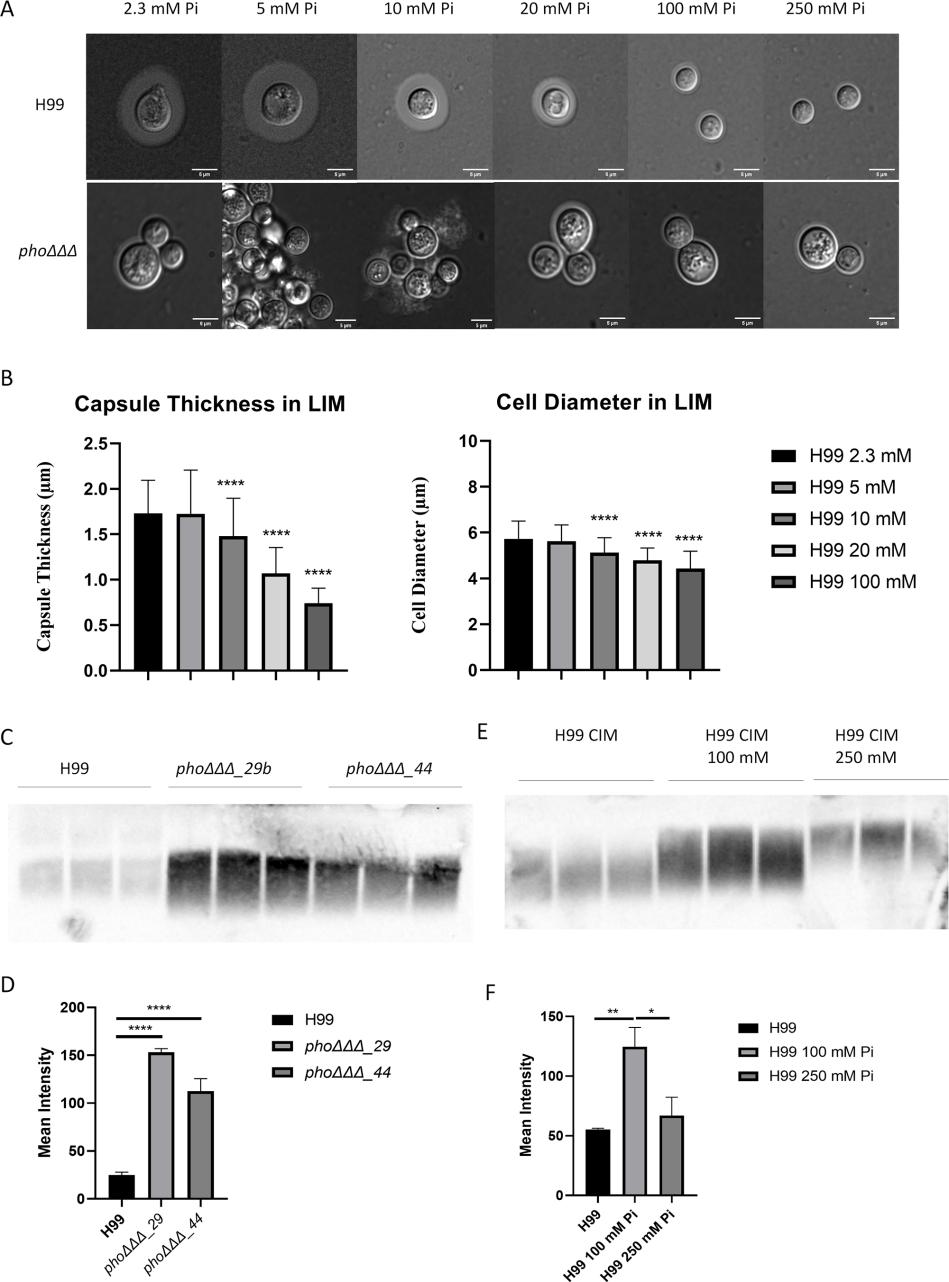
Phosphate suppresses capsule size and promotes polysaccharide shedding. **(A)** Differential interference contrast microscopy (DIC) of capsule stained with India ink for the WT strain (H99) and a *pho*ΔΔΔ mutant grown at 30°C in LIM for 48 h with phosphate levels from 2.3 mM (the level in LIM) to 250 mM, provided as K_2_HPO_4_. (Pi indicates inorganic phosphate, scale bar=5µm) **(B)** Measurement of capsule thickness from **(A)** (n=100, unpaired t test). Three independent experiments were conducted, and the mean and standard deviation of measurement are shown. **(C)** Immunoblot of shed capsule polysaccharide in culture supernatants after 48 h of growth in capsule inducing medium (CIM) for two independent *pho*ΔΔΔ mutants and the WT strain (H99). **(D)** The mean intensity of the shed capsule of each strain from **(C)** was calculated by pixel darkness with ImageJ. **(E)** Immunoblot of shed capsule in culture supernatants after 48 h of growth in CIM in a high phosphate condition of 250 mM Pi. **(F)** The mean intensity of the shed capsule of each treatment from **(E)** was calculated by pixel darkness with ImageJ. Error bar represents the standard deviation. Statistical significance was determined using one-way ANOVA with Tukey’s multiple comparisons in GraphPad Prism 8.0 (GraphPad Software) (*p<0.05 **p<0.01 and ****p<0.0001). For **(C-F)**, three biological replicates are shown, and the assays were repeated three times.

The cell wall is the site of attachment of the polysaccharide capsule via binding to α-1,3-glucan (Reese and Doering, 2003; Crabtree et al., 2012; Srikanta et al., 2014). We previously found that capsule size was reduced in *pho*ΔΔΔ mutants, as was polyphosphate content (Kretschmer et al., 2014). To examine the capsule phenotype in the context of the cell wall changes described above, we tested whether phosphate levels influenced capsule formation for the WT strain and the *pho*ΔΔΔ mutant (**Figures 3A, B**). We first measured orthophosphate levels by molybdate reactivity and confirmed that adding phosphate increased orthophosphate levels for both mutants and WT (**Supplementary Figure S3**). Capsule size was reduced for the WT strain when phosphate exceeded 10 mM and was barely detectable at 100 and 250 mM, whereas *pho*ΔΔΔ mutants showed no response to phosphate and had smaller capsule sizes under all conditions (**Figure 3A**). Cells of the *pho*ΔΔΔ mutant were clustered when grown in 5 mM or 10 mM phosphate as expected for cells with a capsule defect, but this phenotype was not observed at higher concentrations perhaps due to an influence of ionic strength (**Figure 3A**). Addition of phosphate also induced smaller cells in WT but not in the *pho*ΔΔΔ mutant (**Supplementary Figure S3**), a phenotype reported previously (Denham et al., 2022). No growth defect was observed for the WT strain at the concentrations that induced smaller capsule, consistent with our previous study, thus indicating that reduced capsule size was not due to poor growth (Kretschmer et al., 2014). The minimum level of phosphate required for the *pho*ΔΔΔ mutant to grow as well as WT was 12-15 mM, and we found that the highest phosphate level (250 mM) did inhibit the growth of WT (**Supplementary Figure S4**). Another phosphate source in the form of Na_2_HPO_4_ also induced smaller cells and smaller capsules (**Supplementary Figure S3**).

We noted that a small amount of capsule material was present on the cells of the *pho*ΔΔΔ mutant indicating that polysaccharide biosynthesis still occurred, and that impaired attachment might account for the small capsule size. This possibility was examined by assaying the amount of shed capsular polysaccharide in the culture supernatant by immunoblotting. After 48 h of growth, a greater amount of shed capsule was found in the supernatant of the *pho*ΔΔΔ mutants compared with the WT (**Figures 3C, D**). High phosphate (100 mM and 250 mM) also increased the amount of shed polysaccharide for the WT strain, and slower migration of polysaccharides on the gel indicated an influence on the size of the shed material (**Figures 3E, F**). Thus, both impaired phosphate transport in the mutants and exposure to high phosphate for the WT influenced capsule attachment, perhaps reflecting an impact of both conditions on cell wall composition.

### 3.3 Phosphate reduces polysaccharide precursors in capsule-inducing conditions

**Figure 4:**
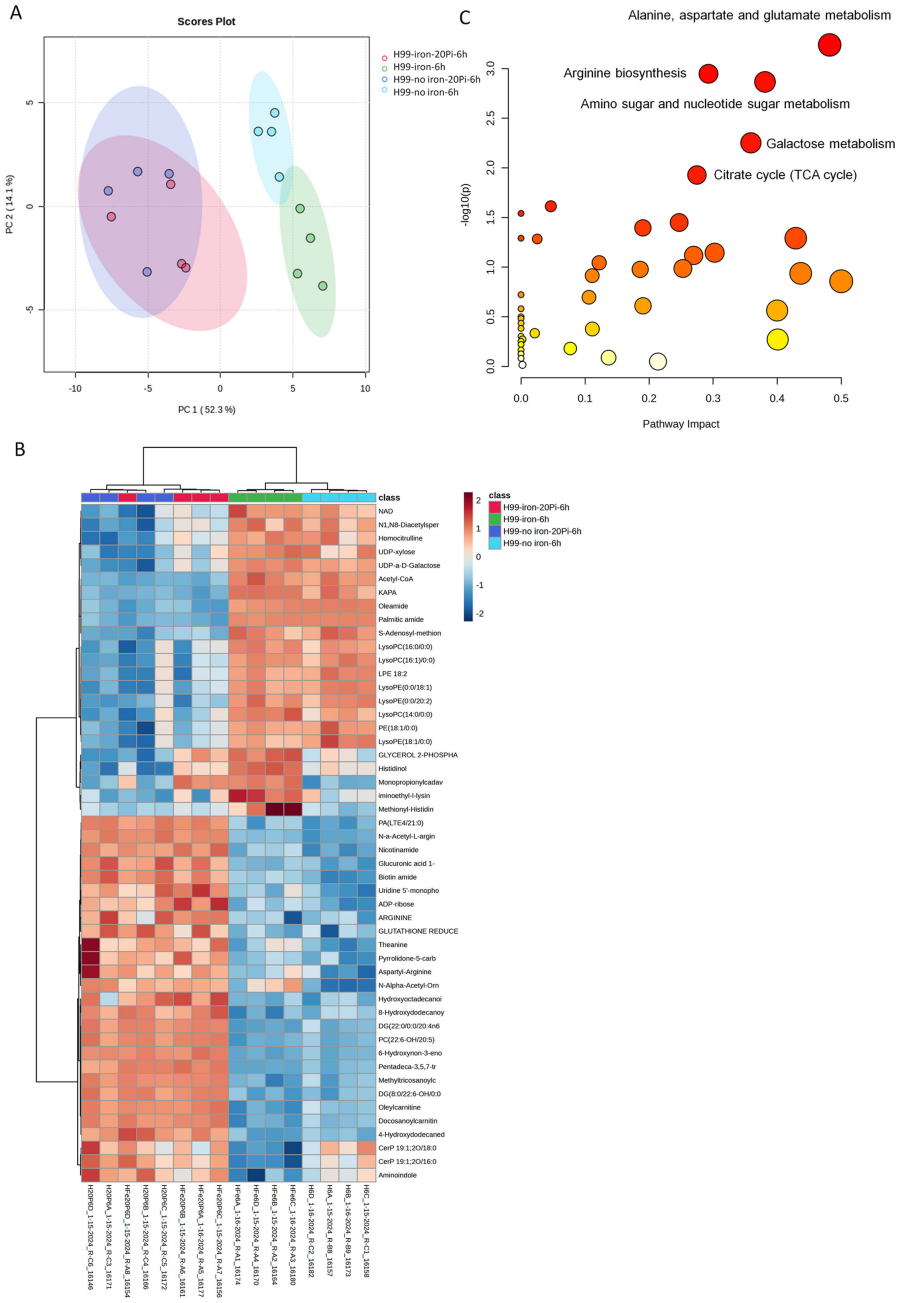
Phosphate treatment alters the metabolic profile of wild-type cells. **(A-C)** LC-HRMS/MS measurements of metabolites for WT cells in high/low phosphate or with/without iron starvation were performed under capsule inducing conditions. **(A)** Multivariate principal-component analysis of clustering of profiles according to sample grouping. **(B)** Heatmap of the analysis for the top 50 compounds of four biological replicates for each sample condition; the red-to-blue scale represents high to low metabolite levels. **(C)** The top five pathways among different groups after LC-HRMS/MS measurements. Plots were generated using MetaboAnalyst 6.0, implementing log transformation and Pareto scaling.

**Figure 5:**
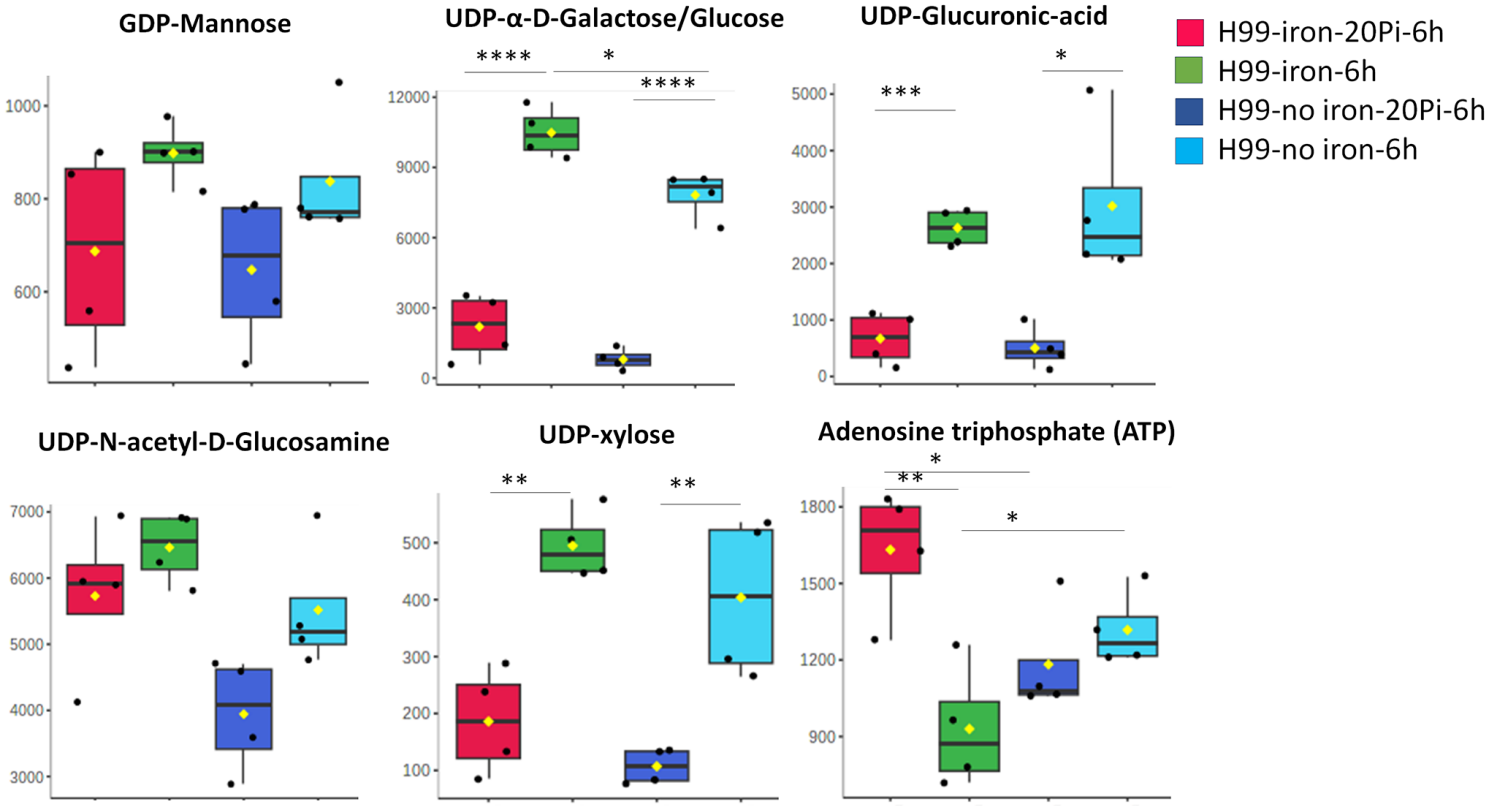
Phosphate induces a reduced level of nucleotide sugars in wild-type cells. Relative metabolite abundance of nucleotide sugars using UDP-α-D-Galactose/Glucose, UDP-xylose, UDP-N-acetyl-D-Glucosamine/Gal, GDP-mannose and UDP-Glucuronic Acid as standards for the measurements (n=4). Cells of the WT were grown in YPD for overnight and then starved in LIM (with iron, low phosphate, 2.5 mM) for 24 h and then transferred in medium with high (20 mM) KH_2_PO_4_ or no iron LIM for 6 h. Cell extracts were subjected to LC-HRMS/MS for untargeted metabolomics. Pi stands for inorganic phosphate. Mean and standard deviation of measurement are shown. Statistical significance was determined using one-way ANOVA with Tukey’s multiple comparisons in GraphPad Prism 8.0 (GraphPad Software) (*p<0.05 **p<0.01 ***p<0.001 and ****p<0.0001).

To examine the impact of phosphate in more detail, we used hydrophilic interaction liquid chromatography-high resolution mass spectrometry (LC-HRMS/MS) to quantify 225 known metabolites in wild-type (WT) cells treated with varying phosphate levels under capsule inducing conditions (CIM). Two different levels of iron were included in the media given the impact of iron on capsule size (Vartivarian et al., 1993). MetaboAnalyst 6.0 was used to identify significantly altered compounds and pathways, and principal component analysis revealed clustering based on phosphate or iron availability (**Figure 4A**). Metabolites measured among different nutrient availability groups were also observed in a heat map with four biological replicates (**Figure 4B**). The top 20 related pathways included nucleotide and nucleobase metabolism, tricarboxylic acid cycle (TCA cycle), galactose, amino acid and amino sugar metabolism (**Figure 4C**, **Supplementary Figure S5A**). We specifically quantified five nucleotide sugars involved in polysaccharide synthesis using UDP-α-D-galactose/glucose, UDP-xylose, UDP-N-acetyl-D-glucosamine, GDP-mannose and UDP-glucuronic acid as standards (**Figure 5**, **Supplementary Figure S5B**). We found that the accumulation of UDP-glucuronic acid, UDP-α-D-galactose/glucose, and UDP-xylose was reduced with phosphate addition regardless of the presence of iron. Differences were not found in the levels of GDP-mannose and UDP-N-acetyl-D-glucosamine. We noted that the level of ATP was higher in abundant phosphate thus indicating the responsiveness of the cells. Overall, these results indicate a potential regulatory influence of abundant phosphate to remodel metabolism. Although a clear pattern with nucleotide sugar abundance did not emerge, we hypothesize that inhibition of capsule elaboration by phosphate occurred in part via an influence on the synthesis of cell wall and capsule polysaccharides in combination with decreased attachment.

### 3.4 The high affinity phosphate transporter Pho840 contributes to tolerance of high calcium levels

**Figure 6:**
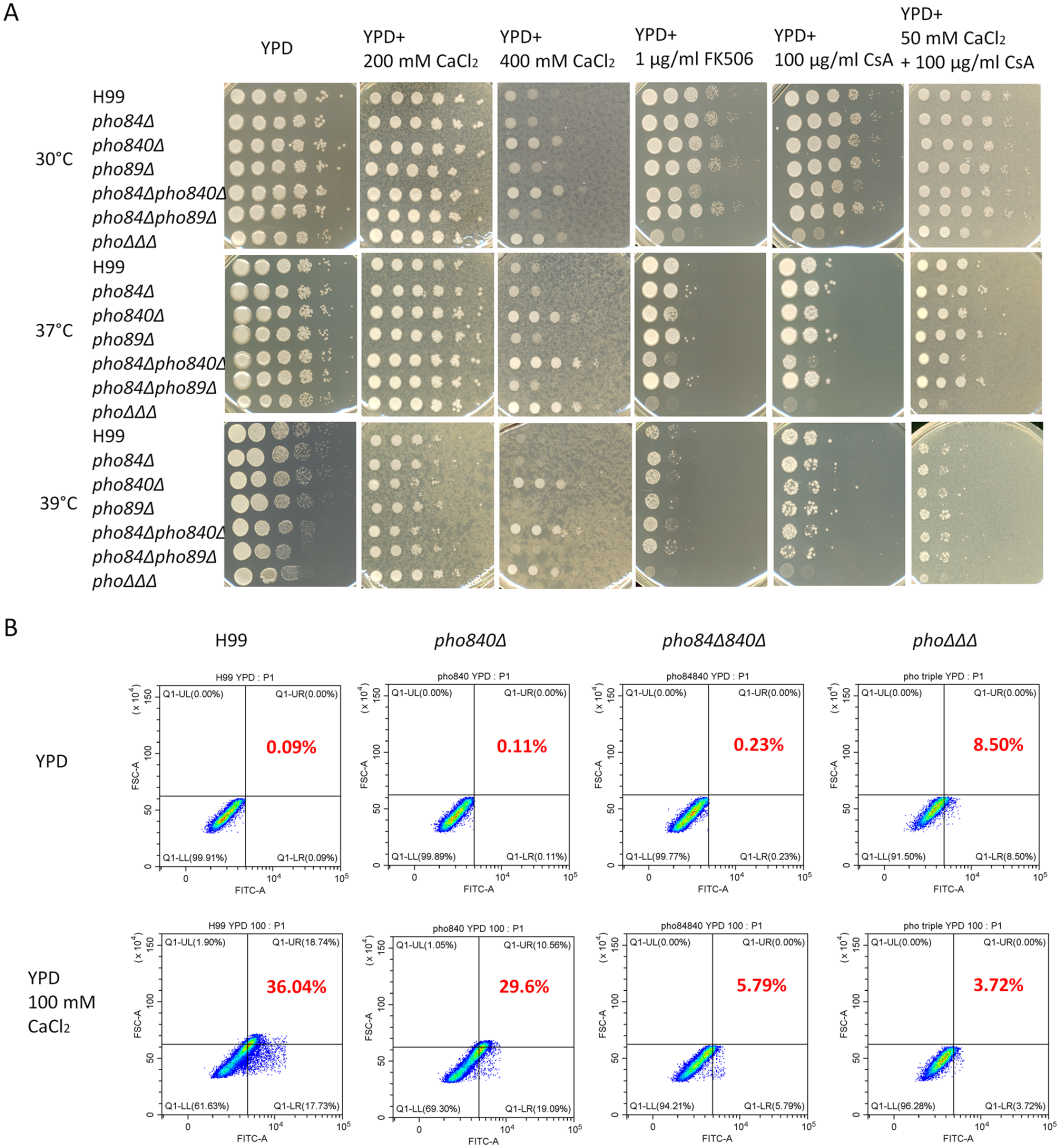
Loss of high-affinity phosphate transporters influences resistance to high calcium and susceptibility to inhibitors of the calcineurin pathway. **(A)** Growth assays for the single, double and triple mutants of the high-affinity phosphate transporters on calcium, inhibitors of the calcineurin pathway, or the calcium chelator EGTA, at the indicated concentrations. The plates were incubated at 30°C, 37°C or 39°C for 2 days and then transferred to 30°C for another 2 days before being photographed. The assays were repeated three times. **(B)** Intracellular calcium in the mutants was assayed with the calcium indicator Cal Green 1 AM (Calcium Green-1 AM) using flow cytometry with the fluorescein isothiocyanate (FITC, 488/530 nm) channel. The assays were repeated three times with three biological replicates each.

Recent findings indicate that caspofungin tolerance is mediated by multiple pathways downstream of calcineurin, and by the RNA-binding protein Puf4 (Pianalto et al., 2019; Kalem et al., 2021). Our previous studies revealed that the *pho*ΔΔΔ mutant had altered susceptibility to calcium and the inhibitor cyclosporine A (CsA) that targets the calcineurin pathway (Kretschmer et al., 2014). We therefore characterized the *pho* transporter mutants with regard to calcium homeostasis and calcineurin inhibition. We found that the *pho840*Δ, *pho84*Δ*840*Δ and *pho*ΔΔΔ mutants showed resistance to high levels of calcium, but susceptibility to the inhibitors FK506 and CsA (**Figure 6A**, **Supplementary Figures S6**, **S7A**). The resistance to calcium was particularly evident at higher temperatures (**Figure 6A**). Furthermore, the growth defect that occurred with the calcineurin inhibitor CsA was partially rescued by adding 50 mM calcium at 30°C (**Figure 6A**). Intracellular Ca^2+^ levels were then measured with the calcium indicator (Calcium Green™-1, AM, cell permeant) and flow cytometry. The WT strain had more calcium after 3 h treatment with CaCl_2_, while the level in the *pho840*Δ, *pho84*Δ*840*Δ and *pho*ΔΔΔ mutants remained low (**Figure 6B**, **Supplementary Figure S7B**). These results suggest that Ca^2+^ influx was disrupted primarily by deletion of *PHO840*.

### 3.5 Mutants lacking high-affinity phosphate uptake are impaired in ER homeostasis

**Figure 7:**
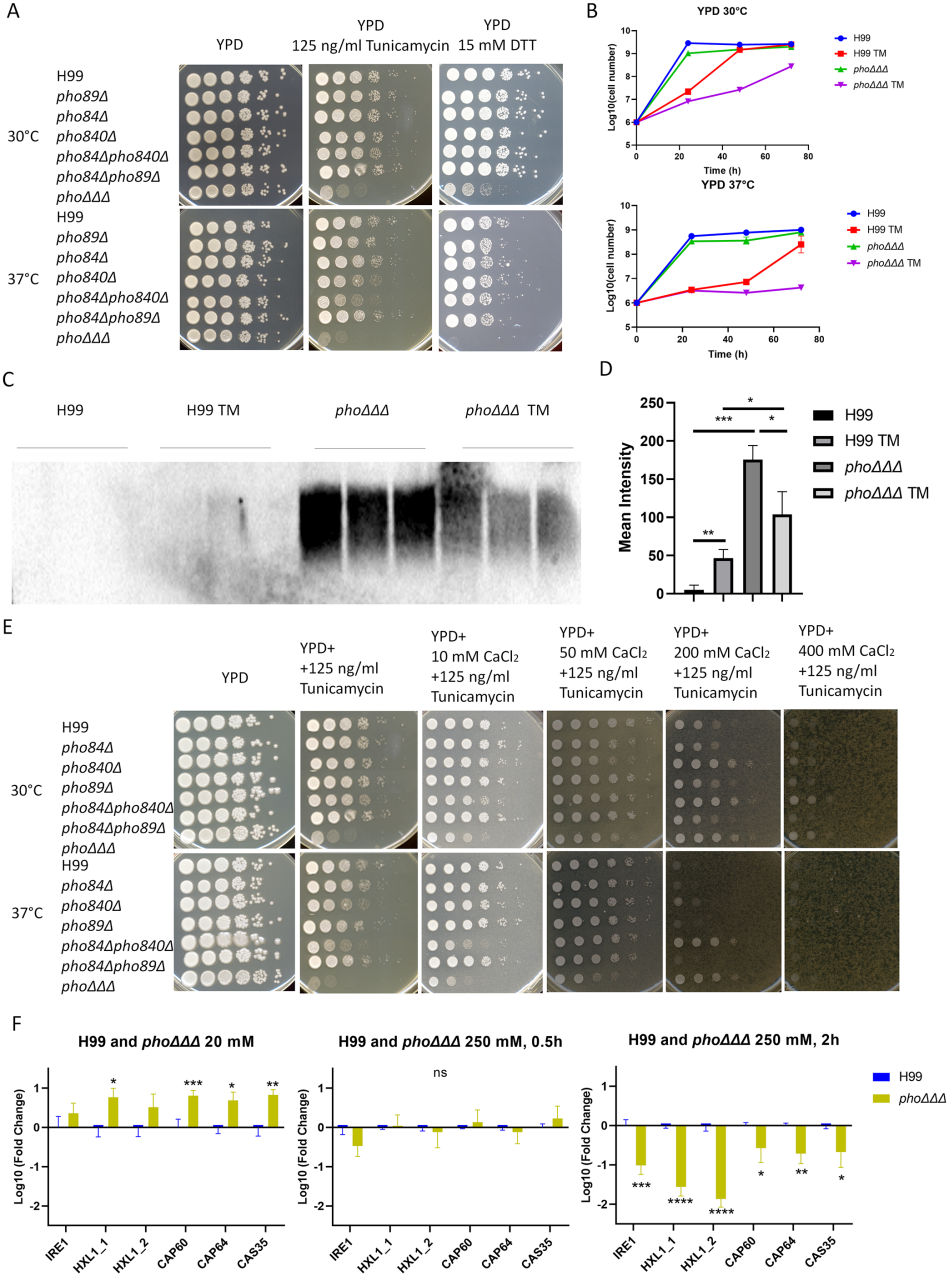
ER function is altered in mutants lacking high-affinity phosphate uptake. **(A)** Growth assays for the indicated mutants in the presence of tunicamycin or dithiothreitol (DTT) to provoke ER stress. Plates were incubated at 30°C, 37°C or 39°C for three days and then photographed. The assays were repeated three times. **(B)** Growth in liquid media for the *pho*ΔΔΔ mutant and the WT strain with and without tunicamycin (TM). Cell numbers were counted every 24 h upon growth at 30°C or 37°C with two replicates for each sample and three repeats of the experiment. **(C)** Immunoblot of shed capsule polysaccharide, detected by 18B7 antibody after 48 h growth in LIM at 30°C. Three biological replicates are shown and three independent experiments were conducted. **(D)** The mean intensity of the shed capsule of each strain from **(C)** was calculated by pixel darkness with ImageJ. **(E)** Growth assays in the presence of tunicamycin alone or in combination with calcium (10 mM, 50 mM, 200 mM and 400 mM). Plates were incubated at 30°C, 37°C and 39°C for 3 days and photographed. The assays were repeated three times. **(F)** qPCR analysis of the transcript levels for genes related to the UPR and capsule synthesis upon growth in low (20 mM) and high (250 mM) phosphate conditions at 0 h, 0.5 h, and 2 (h) The log10 fold changes were compared between WT and the *pho*ΔΔΔ mutant in the different conditions (low phosphate, high phosphate_0.5 h and high phosphate_2 h), with WT as a control. The assays were performed twice with three biological replicates each. The mean and standard deviation of the measurements are shown. Statistical significance was determined by performing with one-way ANOVA and Tukey’s multiple comparisons in GraphPad Prism 8.0 (GraphPad Software). (ns, non-significant; *p<0.05 **p<0.01 and ***p<0.001). Assays were repeated three times.

Given connections between the ER and calcium homeostasis (Groenendyk et al., 2021), we next examined the susceptibility of *pho*ΔΔΔ mutants to agents that provoke ER stress and found increased susceptibility to tunicamycin and DTT, especially at 37°C (**Figure 7A**). These results suggest potential ER dysfunction in the mutants impaired for phosphate uptake. The growth of the *pho840*Δ and *pho84*Δ*pho840*Δ mutants was also slightly inhibited by tunicamycin (**Figure 7A**), suggesting that the Pho84 and Pho840 transporters, but not the Pho89 transporter, may be the main contributors. The *pho*ΔΔΔ mutant had a growth defect with tunicamycin on solid medium and we found the same sensitivity in liquid YPD medium, especially at 37°C (**Figure 7B**). We previously found that tunicamycin reduced capsule size (Geddes et al., 2016), and we therefore quantified shed capsule in culture supernatant with and without tunicamycin treatment to distinguish between an influence on synthesis or attachment. There was no difference for WT, but the amount of shed capsule was significantly reduced in the *pho*ΔΔΔ mutant with tunicamycin, suggesting suppression of capsule synthesis (**Figures 7C, D**). Tunicamycin inhibits protein glycosylation in the ER, which results in the accumulation of misfolded proteins (Banerjee et al., 2011; Wu et al., 2014). Our results with tunicamycin suggest that disruption of phosphate uptake might affect capsule formation by altering glycoprotein biosynthesis, perhaps through an impact of nucleotide sugar precursors (Wang et al., 2018). To further examine calcium homeostasis and ER stress, we added calcium to solid medium containing tunicamycin and found that the growth defect was rescued (**Figure 7E**). Taken together, our results suggest that the phosphate transporters contribute to ER homeostasis.

To further examine the relationship between phosphate and ER homeostasis, we measured the transcript levels for two genes involved in the UPR pathway, *IRE1* and *HXL1* (Cheon et al., 2014). Genes involved in capsule formation (*CAP60, CAP64* and *CAS35*) were also included in our analysis (Janbon, 2004). We used 20 mM for low phosphate and 250 mM for high phosphate to ensure that all cells were in the logarithmic phase of growth, as the *pho*ΔΔΔ mutants were unable to grow under low phosphate conditions (Kretschmer et al., 2014). To ensure that the UPR gene regulation was evaluated in the context of a response to phosphate, we confirmed that transcript levels for the *PHO* regulon genes *PHO84, PHO89, PHO840, PHO81, PHO91*, and *VTC4* were downregulated at 0.5 h and 5 h in the high phosphate condition (**Figure 7F**, **Supplementary Figure S8A**), which is consistent with previous studies (Kretschmer et al., 2014; Toh-E et al., 2015). As expected, we did not detect transcripts for *PHO84, PHO89* or *PHO840* in the *pho*ΔΔΔ mutant (**Figure S8A**). We found that transcript levels for *IRE1* and *HXL1* were upregulated after phosphate addition in the WT strain but downregulated in the *pho*ΔΔΔ mutant; this difference was more dramatic at the time point of 2 h (**Figure 7F**, **Supplementary Figure S8B**). All transcript levels had declined by 5 hours (**Supplementary Figure S8B**). A comparison of the WT strain and the *pho*ΔΔΔ mutants revealed that *IRE1* and *HXL1* were upregulated in *pho*ΔΔΔ mutants in low phosphate, whereas the genes were downregulated after addition of phosphate, as were the capsule-related genes (**Figure 7F**, **Supplementary Figure S8B**). Taken together our results show that high levels of phosphate impact both the UPR and capsule synthesis, and further support a model in which capsule elaboration is insufficient due to a combination of defects in synthesis and attachment at the cell surface.

### 3.6 Phosphate induced a shift from accumulation of triglycerides to phospholipids

**Figure 8:**
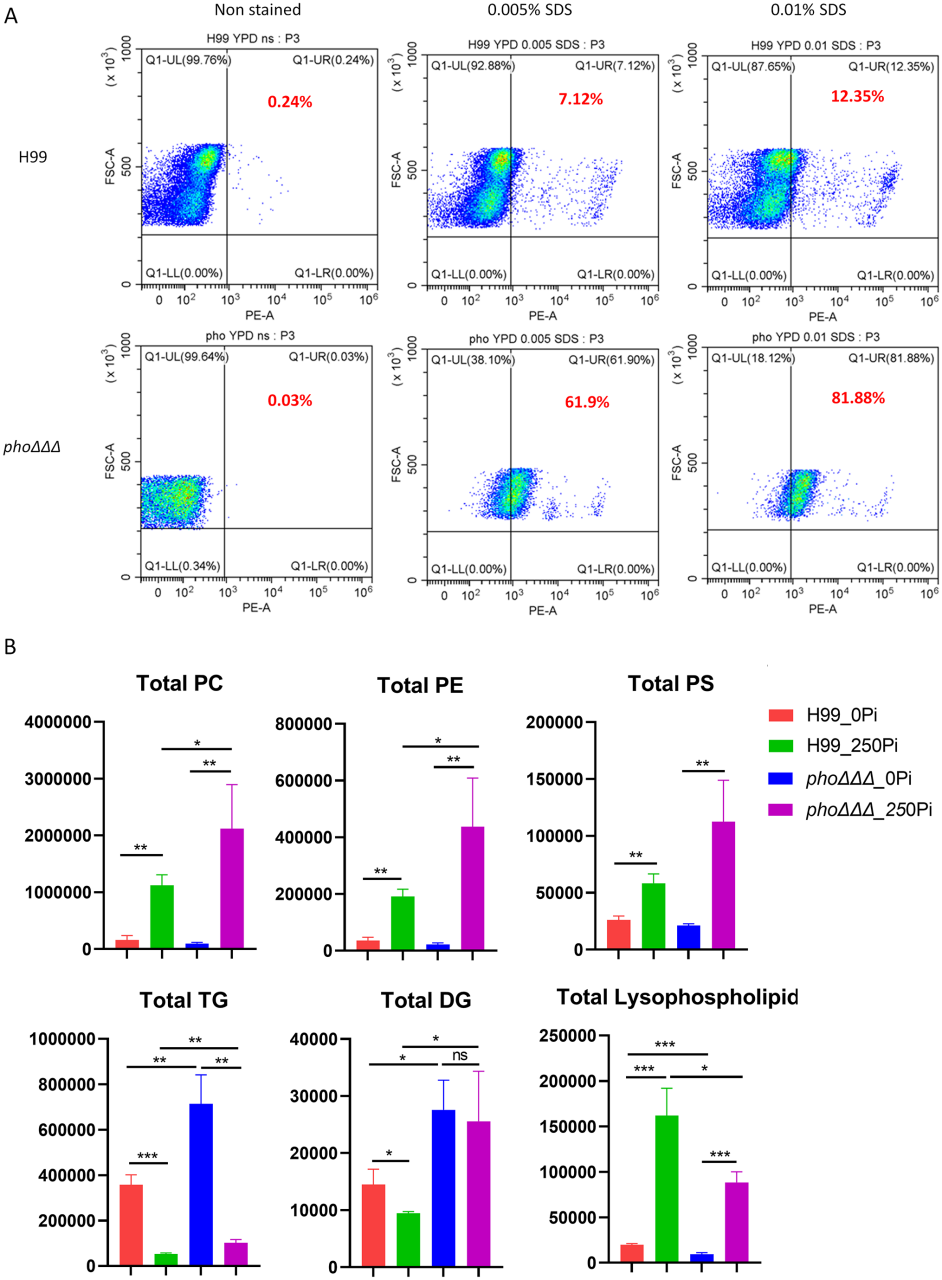
The *pho*ΔΔΔ mutant is susceptible to SDS and has altered lipid composition. **(A)** Flow cytometry assay of cell permeability as tested with 5 μg/ml propidium iodide with and without SDS treatment. The assay was repeated three times with three biological replicates each. **(B)** Comparisons of lipid composition for the WT and the *pho*ΔΔΔ mutant. Three biological replicates were averaged and error bars represent standard deviation. PC: phosphatidylcholine, PE: phosphatidylethanolamine, PS: phosphatidylserine, TGs: triacylglycerols, DGs: diacylglycerols, and Lysophospholipids. Statistical significance was determined using one way ANOVA with Tukey’s multiple comparisons (ns, non-significant; *p<0.05 **p<0.01 and ***p<0.001).

Recent screens of mutant collections identified membrane integrity and phospholipid asymmetry as contributors to caspofungin tolerance (Huang et al., 2016; Hu et al., 2017; Moreira-Walsh et al., 2022). Specifically, the lipid flippase subunit Cdc50 was involved in tolerance to caspofungin, and was also found to regulate calcium homeostasis (Huang et al., 2016; Hu et al., 2017; Cao and Xue, 2020). Furthermore, caspofungin tolerance has been linked with altered membrane permeability (Moreira-Walsh et al., 2022). In this context, we found that *pho*ΔΔΔ mutants failed to grow on 0.01% SDS plates at a range of temperatures, but no difference was found in the single or double deletion mutants (**Supplementary Figure S1A**). The *pho*ΔΔΔ mutants were also sensitive to agents that caused osmotic stress such as 1.5M KCl, 1.5M NaCl and 1.5 M sorbitol (**Supplementary Figure S1A**). We therefore examined differences in membrane permeability in more detail with propidium iodide (PI) staining. After 3 h incubation with SDS (0.01% and 0.005%), the staining intensity for the mutant *pho*ΔΔΔ shifted dramatically thus indicating greater permeability (**Figure 8A**). Taken together, our results indicated that disruption of phosphate uptake system leads to impaired plasma membrane integrity.

We next examined changes in lipid composition in response to phosphate starvation. The WT strain and a *pho*ΔΔΔ mutant were incubated in minimal medium for 24 h in the presence or absence of phosphate, and total lipids were extracted and analyzed by LC-HRMS/MS. Phosphate treatment increased phospholipids and decreased triglycerides (TGs) both in the WT strain and the *pho*ΔΔΔ mutant. The shift was more dramatic in the mutant with more TGs in the phosphate starvation condition and more phospholipids with phosphate repletion, compared with WT (**Figure 8B**). We further quantified the total amount of phosphatidylcholine (PC), phosphatidylethanolamine (PE), phosphatidylserine (PS), triglycerides (TGs), diacylglycerols (DGs) and lysophospholipids (LPLs), the major phospholipids found in *C. neoformans* membranes as identified previously (Carman and Han, 2011; Singh et al., 2017; Lev et al., 2019). We found significantly increased phospholipids in high phosphate conditions, including PC, PE, PS and lysophospholipids in both WT and mutant (**Figure 8B**, **Supplementary Figure S9**). However, PC, PE, and PS were lower in the mutant compared to WT, while the opposite was true for lysophospholipids. There was a large accumulation of TGs in phosphate starvation conditions in both the WT strain and the *pho*ΔΔΔ mutant, and the mutant had more TGs, compared with WT. DGs largely declined with sufficient phosphate in WT but not in the *pho*ΔΔΔ mutant. Overall, our results revealed a shift in lipid composition in response to defects in phosphate uptake. The observed changes in phospholipids may contribute to the impact of phosphate on capsule via trafficking or the activity of membrane-associated enzymes, or given the observation that phospholipids induce capsule enlargement (Chrisman et al., 2011).

### 3.7 Cell size is influenced by high-affinity phosphate uptake *in vitro* and *in vivo*

**Figure 9:**
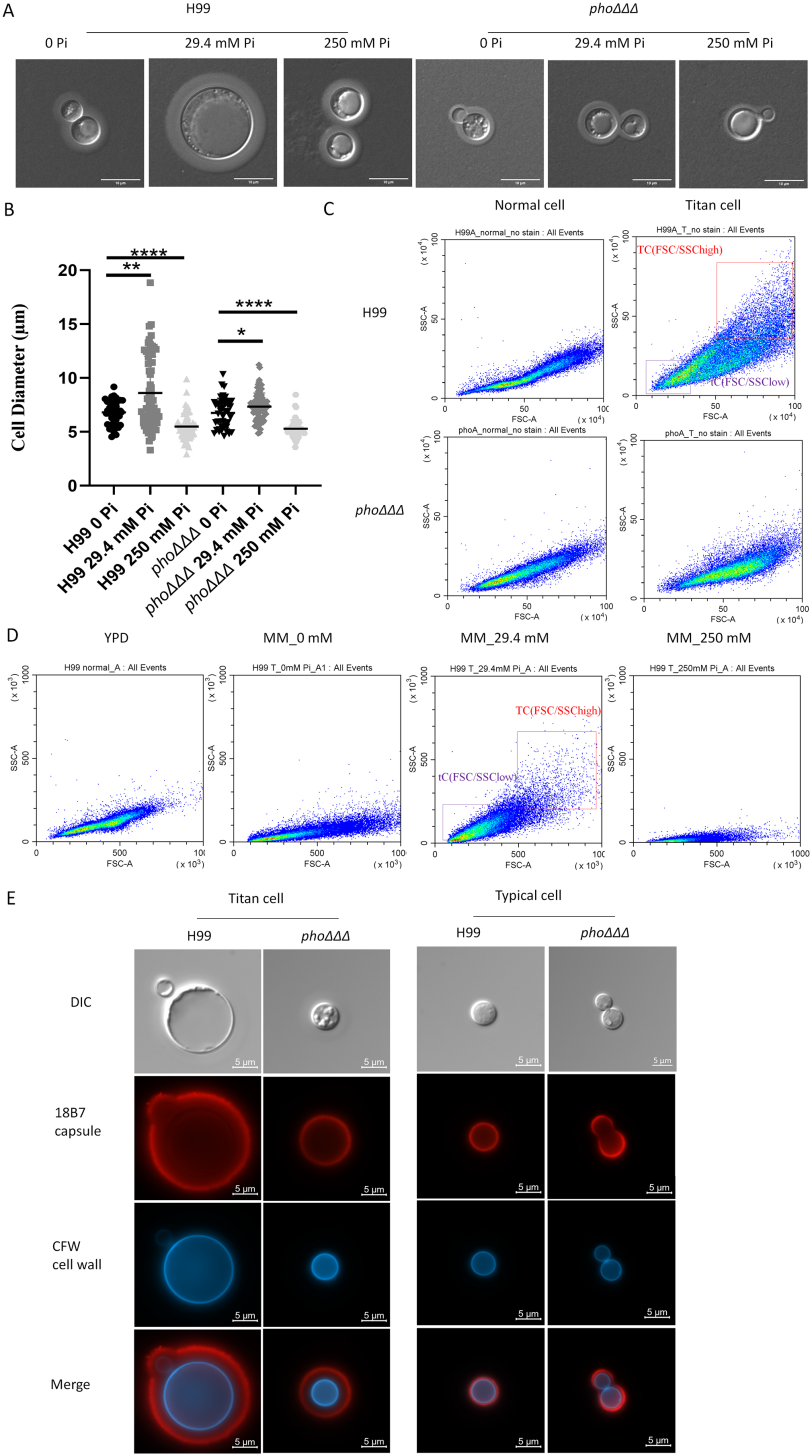
The *pho*ΔΔΔ mutant does not form titan cells *in vitro* **(A)** DIC images of cells of the WT strain H99 and the *pho*ΔΔΔ mutant stained with India ink to reveal capsule size relative to cell size in response to the indicated levels of phosphate. Scale bar=10 µm. **(B)** Cell sizes from the experiment in **(A)** were measured and analyzed by ImageJ. Error bars represent standard deviation. Statistical significance was determined using one-way ANOVA with Tukey’s multiple comparisons in GraphPad Prism 8.0 (GraphPad Software) (*p<0.05 **p<0.01 and ****p<0.0001). **(C)** Flow cytometry dot plots (FSC/SSC) to compare the WT strain H99 and *pho*ΔΔΔ mutant under conditions to induce titan cell formation *in vitro* with flow cytometry; FSC/SSChigh and FSC/SSClow represent titan cells (TC) and typical cells (tC), respectively. The assays were repeated three times. **(D)** Flow cytometry dot plots (FSC/SSC) to measure cell size in YPD and the indicated levels of phosphate. Three independent experiments were performed. **(E)** DIC and fluorescence images of cells from titan cell inducing medium and YPD rich media taken with DAPI (CFW staining) and Alexa fluor 568 (18B7 antibody) channels for chitin and capsule, respectively. (Red=capsule, blue=chitin) (scale bar=5µm). Three independent experiments were performed.

The formation of enlarged and small cells by *C. neoformans* is a key aspect of the infection process (Crabtree et al., 2012; Denham et al., 2022). Enlarged cells that are greater than 10 μm in diameter are called titan cells while normal cells are ~5 μm in diameter. To test whether loss of the high-affinity phosphate transporters affects titan cell formation *in vitro*, cells were grown in minimal media with different phosphate concentrations (0, 29.4 and 250 mM) at 30°C for 48 h, as previously described (Kretschmer et al., 2014). We found that WT cells produced titan cells with 29.4 mM phosphate, not in the 0 mM or 250 mM conditions, while the *pho*ΔΔΔ mutant did not form titan cells in all three conditions, indicating a dependence on phosphate uptake (**Figure 9A**). No defect in titan cell formation was found for the single mutants (**Supplementary Figure S10A**). The WT cells in 29.4 mM phosphate had the largest cell size compared to all other conditions, and high phosphate decreased the cell size (**Figure 9B**). Analysis of cell size by fluorescence activated cell sorting (FACS) using a published protocol (Hommel et al., 2018) revealed that the WT cells shifted to larger sizes in MM, as more cells were located at the positive side of FSC-A/SSC-A while no difference was found for the *pho*ΔΔΔ mutants (all of the cells remained small) (**Figure 9C**). The FACS analysis of WT cells in MM media with different phosphate levels (0, 29.4 mM and 250 mM) also showed that high phosphate induced smaller cells (**Figure 9D**), which is consistent with the microscopic observations (**Figure 9A**). Further microscopy confirmation of differences in titan cell formation was obtained by staining WT and *phoΔΔΔ* mutant cells with the anti-capsule antibody 18B7 conjugated to Alexa Fluor 568, and with CFW to detect chitin on the cell wall (**Figure 9E**). Taken together, our results indicate a role for phosphate homeostasis in titan cell formation.

**Figure 10:**
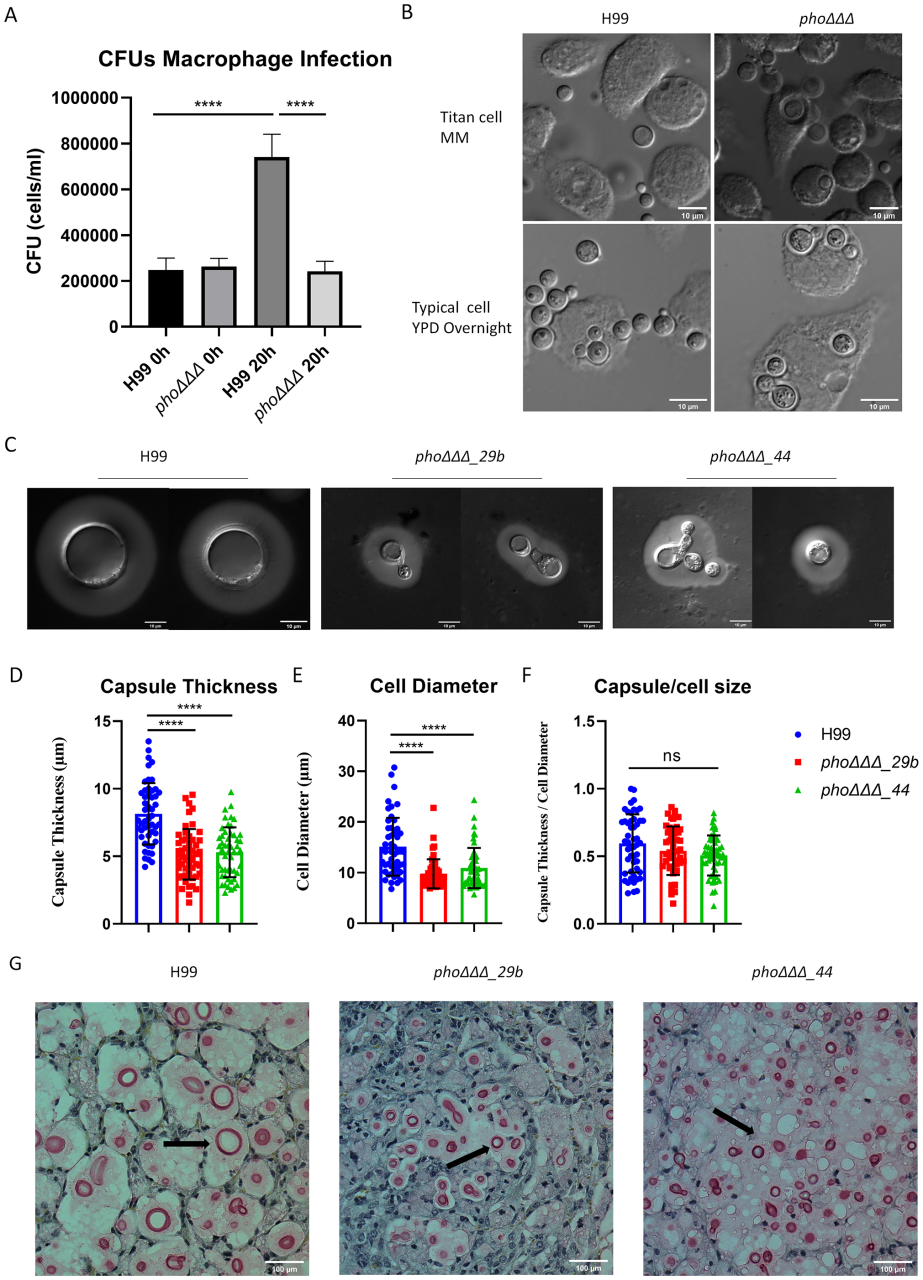
The *phoΔΔΔ* mutant does not form titan cells in murine tissue. **(A)** Phagocytosis (after 2 h of infection) and proliferation (20 h after phagocytosis) of the WT strain and the *pho*ΔΔΔ mutant in the macrophage-like cell line J774.A1. The assays were repeated three times. **(B)** Differential interference contrast microscopy (DIC) of phagocytosed cells (2 h of infection) using titan cells or cells grown in YPD. Scale bar = 10µm. **(C)** Differential interference contrast microscopy (DIC) of WT and mutants (*pho*ΔΔΔ*_29b* and *pho*ΔΔΔ*_44*), collected from infected mice at the experimental end point (>15% weight loss). Scale bar=10 µm. **(D-F)** Measurements of capsule thickness **(D)**, cell diameter **(E)** and the ratio of capsule thickness/cell diameter **(F)** for cells collected from infected mice (n=50, error bars represent standard deviation). Statistical significance was determined by performing one-way ANOVA and Tukey’s multiple comparisons in GraphPad Prism 8.0 (GraphPad Software). (ns, non-significant; ****, P < 0.0001). **(G)** Histopathology of the lungs from the experiment shown in **(C)**. Lungs were fixed and stained with mucicarmine. Scale bar =100 μm. Arrows indicate *Cryptococcus* cells.

To further examine the influence of cell size for the *pho*ΔΔΔ mutant, we first examined the ability of the J774A.1 macrophage-like cell line to phagocytose cells of the *pho*ΔΔΔ mutant. We found that the mutant had the same phagocytosis rate as WT, but much less proliferation after 20 h (**Figure 10A**, **Supplementary Figures S10B, C**). We also noted that cells of WT grown in titan cell-inducing media were less phagocytosed by macrophages, compared with *pho*ΔΔΔ mutants (**Figure 10B**). Our previous analysis of disease revealed that the *pho*ΔΔΔ mutant was attenuated in virulence but still caused disease and had similar fungal burden as WT in lungs and brains (Kretschmer et al., 2014). We repeated the mouse infection assay to determine whether the *pho*ΔΔΔ deletion also influenced cell size *in vivo* (**Supplementary Figures S10D, E**). We observed an influence of the mutation on survival as seen previously, and we recovered cells from infected lungs to measure and compare the sizes of *pho*ΔΔΔ mutants and WT cells (**Figures 10C-F**, **Supplementary Figures S10D, E**). The cell size of the *pho*ΔΔΔ mutants was much smaller than WT, as was capsule thickness, but no difference was found in the ratio of capsule/cell diameter (**Figures 10D-F**). Histopathology of lung tissues further confirmed the *pho*ΔΔΔ mutants had smaller cells and smaller capsule than WT (**Figure 10G**). Taken together, our results establish a link between phosphate homeostasis and titan cell formation in early infection, and confirm the influence of phosphate on the small cell morphotype, a phenotype that may contribute to dissemination (Denham et al., 2022).

## 4 Discussion

The physiological factors that regulate virulence factor elaboration in *C. neoformans* are poorly understood. We previously discovered that a *pho*ΔΔΔ mutant lacking the three high-affinity phosphate transporters was defective in formation of the polysaccharide capsule that is a major virulence factor (Villena et al., 2008; Zaragoza et al., 2008; Kretschmer et al., 2014). Synthesis of capsule polysaccharide is thought to occur intracellularly and involve nucleotide sugar metabolism, transporters, and glycosyltransferase activities (Wang et al., 2018). An intersection with phosphate homeostasis occurs because nucleotide sugars (e.g., GDP-mannose, UDP-galactose) are the substrates for synthesis of the capsule polysaccharides (Li et al., 2017; Wang et al., 2018; Guazzelli et al., 2020). Nucleotide sugars are also critical for the synthesis of cell wall polysaccharides including glucans and chitin (Gow and Lenardon, 2023). Interestingly, high phosphate influenced cell wall composition and induced a decrease in the accumulation of some nucleotide sugars (e.g., UDP-xylose) in the WT under capsule-inducing conditions. We speculate that abundant phosphate may exert a regulatory influence on the expression or activity of enzymes needed for the synthesis of some nucleotide sugar precursors. Certainly, an impact on nucleotide sugars is consistent with our observation that high phosphate inhibits capsule formation. It is also possible that elevated phosphate could influence nucleotide sugar transport or the function of specific cellular compartments to impair capsule formation (e.g., polysaccharide synthesis in the ER and/or Golgi, and export). Additionally, cells may differentially regulate the biosynthetic processes for cell wall versus capsule formation in response to phosphate availability. Further studies are needed to more fully understand the influence of abundant phosphate on polysaccharide synthesis.

Caspofungin inhibits β-1,3-glucan synthesis and is effective against *Aspergillus* and *Candida* species but not *C. neoformans* (Kartsonis et al., 2003; Formosa et al., 2013; Walker and Munro, 2020; Papon and Goldman, 2021; Mukaremera, 2023). The decreased tolerance of the *pho*ΔΔΔ mutant indicated a change in cell wall structure, especially with regard to glucan synthesis. In this context, α-1,3-glucan is critical for the attachment of capsule polysaccharide (Reese and Doering, 2003). Thus, it is likely that phosphate limitation influenced the availability of nucleotide sugar precursors, impairing the synthesis of cell wall polysaccharides and conditioning both reduced capsule attachment and caspofungin tolerance. This conclusion is consistent with the impact of phosphate limitation on nucleotide sugar precursors for cell wall synthesis previously demonstrated for a mutant lacking Pho84 in *Candida albicans* (Liu et al., 2020). Liu et al. found that the *pho84* mutants had lower nucleotide sugar but higher nucleobase and nucleoside content than WT cells in the presence of phosphate. Furthermore, chitin levels in the cell wall of *C. albicans* are known to increase in response to caspofungin (Wagner et al., 2023). Our analyses revealed differences in staining for cell wall components (chitin and glucans) in the *pho*ΔΔΔ mutant. These differences may reflect compositional changes and/or altered access of the stains due to the absence of the capsule. More detailed quantitative analyses will be needed to fully understand cell wall changes.

Caspofungin tolerance in *C. neoformans* also involves functions that influence membrane composition (Huang et al., 2016; Hu et al., 2017). Specifically, Huang et al. discovered that a mutant lacking *CDC50* was sensitive to caspofungin as well as fluconazole, SDS and KCl. Cdc50 is the noncatalytic subunit of type IV P‐type ATPases (flippases) that regulate bilayer asymmetry, and tolerance suggests that defects in the plasma membrane may influence the intracellular accumulation of caspofungin. Changes in the plasma membrane permeability were also implicated in caspofungin tolerance in a mutant screen conducted by Moreira-Walsh et al. (2022). This study identified mutants in ergosterol biosynthesis and sterol transport with decreased tolerance. Interestingly, we found that the *pho*ΔΔΔ mutant shared the above phenotypes with the *cdc50* mutant suggesting an impact of the plasma membrane due to insufficient phosphate uptake. In particular, 0.01% SDS completely inhibited the growth of the *pho*ΔΔΔ mutant and increased cell permeability. Sensitivity to SDS is therefore a shared trait with both the *cdc50* mutant and with the mutants with identified by Moreira-Walsh et al. (2022).

Consistent with the association between membrane changes and caspofungin sensitivity, our analysis of lipids in the WT and the *pho*ΔΔΔ mutant with and without phosphate revealed a significant shift between phospholipids and TGs. The shifts were more dramatic in the *pho*ΔΔΔ mutant with higher levels of TGs and DGs under phosphate limitation conditions compared with higher PC, PE and PS levels in phosphate rich condition. Phospholipid remodeling in response to phosphate starvation has been studied in detail for *S. cerevisiae, C. albicans, C. neoformans* and in some bacteria (Geske et al., 2013; Riekhof et al., 2014; Yadav et al., 2015; Lev et al., 2019; Köhler et al., 2020; Lejeune et al., 2021; King et al., 2023). In *S. cerevisiae*, the low phosphate condition induces an increase in non-polar lipids (TAG and DAG) and this condition is regulated by Pho4, the key transcription factor for the phosphate regulon (Yadav et al., 2015). Upon phosphate starvation in *C. neoformans*, phosphorus-free betaine lipids (DGTS and DGTA) are found in WT instead of phospholipids (PC, PE and PS), and this transition was disrupted by loss of the Pho4 regulator (Lev et al., 2019). A *pho4*Δ mutant had more PC upon phosphate limitation compared with WT, but differences in PE and PS levels were not observed (Lev et al., 2019). Notably, Pho4 is also involved in cell wall integrity for *C. neoformans* under phosphate limiting conditions (Lev et al., 2017). There may be other connections between phosphate and capsule formation because previous studies found that purified phospholipids (PG, PA, PE, PG, PI, PS and lysophosphatidylcholine (LC)) induce enlarged capsules in *C. neoformans* (Chrisman et al., 2011).

Our current and previous analyses of the phosphate transporter mutants revealed that the *pho840*Δ*, pho84*Δ*840*Δ and *pho*ΔΔΔ mutants were more resistant in high levels of calcium but more susceptible to inhibitors FK506 and CsA that target calcineurin (Kretschmer et al., 2014; Chow et al., 2017). These findings are consistent with other studies that revealed connections between caspofungin tolerance and the calcineurin pathway in *C. neoformans* (Del Poeta et al., 2000; Pianalto et al., 2019; Papon and Goldman, 2021). For example, Kalem et al. (2021) found that the pumilio/FBF RNA binding protein family member Puf4 influenced caspofungin tolerance by binding to and regulating the stability of the transcript for *FKS1* encoding β-1,3-glucan synthase. Additionally, Pianalto et al. (2019) identified a cnb1Δ mutant lacking the calcineurin B regulatory subunit as well as mutations in functions for cell wall biosynthesis that conferred caspofungin sensitivity. They concluded that caspofungin tolerance was conditioned by multiple pathways downstream of calcineurin signaling. Thus, the decrease in caspofungin tolerance for our *pho*ΔΔΔ mutant may also reflect an impact on signaling pathways as well as a contribution to cell wall biosynthesis. Similarly, our analysis revealed connections with calcium and ER stress for our phosphate transporter mutants. We found that cellular calcium levels were high in the WT strain but lower in the *pho840*Δ, *pho84*Δ*pho840*Δ and *pho*ΔΔΔ mutants. Loss of Pho840 in particular led to an imbalance in intracellular calcium homeostasis. We noted that the *pho840*Δ*, pho84*Δ*pho840*Δ and *pho*ΔΔΔ mutants had a growth defect on the protein glycosylation inhibitor tunicamycin but were rescued by the inclusion of calcium in the medium. ER stress caused by tunicamycin is also linked to calcium homeostasis in *S. cerevisiae* and this is consistent with the role of the ER as the main Ca^2+^ storage organelle (Bonilla and Cunningham, 2003; Dudgeon et al., 2008; Groenendyk et al., 2021). ER stress provokes the unfolded protein response (UPR) and connections between the UPR pathway and Ca^2+^ homeostasis have been studied in *Aspergillus fumigatus* (Weichert et al., 2020), *Candida glabrata* (Miyazaki and Kohno, 2014) and *C. neoformans* (Cheon et al., 2011; Jung et al., 2018). Given the observed impact of the *pho*ΔΔΔ mutation on phospholipid composition, we note that many genes encoding enzymes for lipid synthesis are upregulated upon UPR activation (Travers et al., 2000; Jonikas et al., 2009; Thibault et al., 2012; Volmer et al., 2013). Lipid imbalance also disrupts calcium homeostasis in mammals and perturbed calcium metabolism can activate the UPR pathway by accumulation of fatty acids (Fu et al., 2011; Cui et al., 2013). Finally, it is known that divalent cations also influence capsule assembly at the cell surface, e.g., Ca^2+^ depletion by EDTA reduces capsule size and increases shedding (Nimrichter et al., 2007).

We also examined cell size for the *pho*ΔΔΔ mutant to explore underlying reasons why the mutant was attenuated for virulence yet the fungal burden of the mutant was similar to WT in lung or brain tissue (Kretschmer et al., 2014). Morphological shifts are an important aspect of the interaction of *C. neoformans* with host tissue. For example, cells isolated from the lung have larger capsules than cells from the brain (Charlier et al., 2005). Conditions relevant to the host can also stimulate capsule enlargement *in vitro* and these include low iron, high CO_2_, and the presence of serum (Zaragoza and Casadevall, 2004). Additionally, a subpopulation of enlarged (titan) cells (> 10 μm in diameter) appear during lung infection (Granger et al., 1985; Vartivarian et al., 1993; Okagaki et al., 2010; Zaragoza et al., 2010; Crabtree et al., 2012; Okagaki and Nielsen, 2012; Hommel et al., 2018; Dyląg et al., 2020).Titan cells also have altered capsule structure, a thick cell wall, higher resistance to oxidative and nitrosative stresses, the ability to avoid phagocytosis, and polyploidy resulting from endoreduplication. Our results indicated that the *pho*ΔΔΔ mutant did not form titan cells *in vitro* or *in vivo*. We also noticed that both low (0 Pi) or high (250 mM Pi) phosphate impaired titan cell formation compared to an intermediate level (29.4 mM Pi). The connections of phosphate and cell size were studied previously in *C. neoformans* with the observation that phosphate induced smaller cell sizes, which facilitate the dissemination to the brain (Sorrell et al., 2016; Denham et al., 2022). Loss of the phosphate regulator Pho4 also caused a smaller cell size in infected lung tissue (Lev et al., 2017).

**Figure 11:**
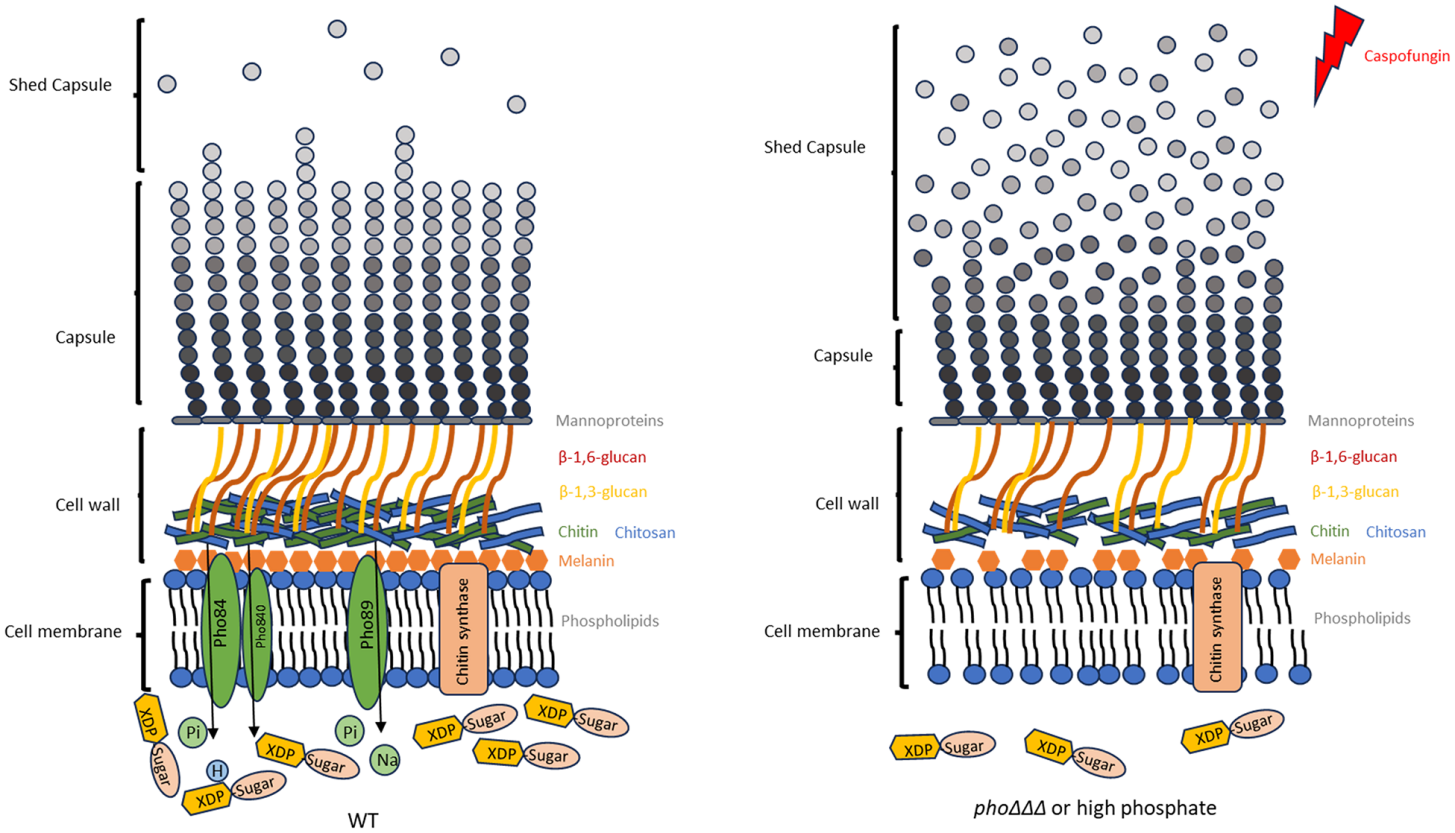
Model for the role of phosphate in cell wall structure and capsule attachment. Polysaccharide capsule, cell wall and cell membrane are shown for the WT strain and the mutant lacking all three high affinity phosphate transporters or WT under high phosphate conditions. We propose that deletion of high-phosphate transporters results in reduced capsule formation by affecting polysaccharide biosynthesis via an influence on nucleotide sugar metabolism. Additionally, this influence affects the cell wall composition as indicated by a change in caspofungin tolerance and this impairs the attachment of polysaccharide to cell wall. Decreased chitin, melanin formation and changes in phospholipid composition affected by phosphate are thought to be responsible for the observed defects in cell wall structure and increased capsule shedding, reduced resistance to caspofungin and ultimately the reduced virulence of the mutants.

In summary, we have uncovered the influence of phosphate on cell wall structure and attachment of capsule polysaccharide, a key virulence factor in cryptococcosis (**Figure 11**). This work adds a new level of complexity to the physiological conditions that regulate virulence in *C. neoformans*. Our findings also contribute to a growing appreciation of the variety of factors that condition caspofungin tolerance in *C. neoformans.* This appreciation and insights into the physiology of virulence may yield therapeutic strategies to exploit new drug targets and employ combinations of drugs to overcome caspofungin tolerance.

## Author’s note

The content is solely the responsibility of the authors and does not necessarily represent the official views of the National Institutes of Health.

## Data Availability

The original contributions presented in the study are publicly available in the Metabolomics Workbench (Sud et al., 2016). This data can be found here: https://dx.doi.org/10.21228/M8TN7J (project PR002033).

## Supplementary material

Supplementary Figure 1 The *pho*ΔΔΔ mutant is sensitive to SDS, Congo red, osmotic stress and fluconazole. **(A)** Assays to compare the growth of the WT strain and the *pho*ΔΔΔ mutant on media with SDS, agents that challenge cell wall integrity (caffeine, calcofluor white (CFW), and congo red), osmotic stress agents (KCl, NaCl, sorbitol) and antifungal drugs. **(B)** Spot assay showed defects in the complete set of mutants defective in transporters for growth under ER (125 ng/ml tunicamycin), plasma membrane (0.01% SDS), salt (1.5M KCl, 1.5M NaCl) and nitrosative stressors (NaNO_2_). **(C)** Spot assays of the *pho*ΔΔΔ mutant upon growth in the presence of zinc, nickel or rapamycin at the indicated concentrations.

Supplementary Figure 2 The *pho*ΔΔΔ mutant has an altered cell wall. **(A)** Levels of chitin (CFW) and chitosan (eosin Y) determined by flow cytometry with Pacific Blue (PB450, 405/450 nm) and fluorescein isothiocyanate (FITC, 488/530 nm), respectively. Cells were pre-grown in YPD and then transferred into minimal media (YNB, with different phosphate levels) for 24 h and the assays were repeated three times with three biological replicates each. ΔMFI = change in mean fluorescence intensity. Error bars represent standard deviation. (B,C) WT and mutant were tested in 1:2 serial dilution of three drugs: nikkomycin, manumycin and foscarnet in YNB medium containing 2% glucose and 100mM phosphate provided as KH_2_PO_4._ 96 well plates were incubated at 30°C **(B)** and 37°C **(C)** up to 72 hours and OD_600_ were recorded every 24 hours. Assays were repeated three times. The heat map in **(B)** shows the ratio calculated from the OD measurements of **Figure 2B**, that is, the ratio of treatment with drug to treatment without drug.

Supplementary Figure 3 Phosphate influences β-glucan levels, cell size and capsule thickness in the wild type strain H99. **(A)** Levels of β-glucan were determined by flow cytometry with aniline blue staining (PB450, 405/450 nm). Cells were pre-grown in YPD and then transferred into minimal media (YNB, with different phosphate levels and buffered with 50 mM MOPS) for 24 h and the assays were repeated two times with three biological replicates each. ΔMFI = change in mean fluorescence intensity. Error bars represent standard deviation. **(B)** Orthophosphate levels were measured by molybdate reactivity. Cells were grown in YPD for overnight and transferred into YNB with different phosphate levels for 24h. OD700 nm were calculated based on standard curves generated from the phosphoric acid standards. (n=3) **(C)** Measurements of cell diameter of the WT strain H99 and the *pho*ΔΔΔ mutant at 30°C LIM after 48 h with different levels of phosphate provided as KH_2_PO_4_. (n=50) **(D)** measurement of capsule thickness, cell size and the ratio of capsule thickness/cell diameter of H99 at 30°C LIM after 48 h with phosphate provided as NaH_2_PO_4_. (n=50). Three independent experiments were conducted, mean and standard deviation were shown, statistical significance was determined by performing with one-way ANOVA and Tukey’s multiple comparisons in GraphPad Prism 8.0 (GraphPad Software) (ns, non-significant; *p<0.05 **p<0.01 and ***p<0.001).

Supplementary Figure 4 The *pho*ΔΔΔ mutant has a growth defect in the phosphate limiting condition. WT and mutant were tested in a 1:2 serial dilution of phosphate levels starting with 50 mM and 250 mM Pi, phosphate provided as KH_2_PO_4._ An initial level of 10^5^ cells was added into each well with a media volume of 200 μl. 96 well plates were incubated at 30°C up to 72 h and OD_600_ readings were recorded every 24 h. Three independent experiments were performed with three biological replicates each.

Supplementary Figure 5 Phosphate influences the metabolome. **(A)** The top 20 pathways for metabolites in the WT strain in low/high phosphate or with/without iron conditions for 6 h, as measured by LC-HRMS/MS. (n=4) Cells of the WT were grown in YPD for overnight and then starved in LIM (with iron, low phosphate, 2.5 mM) for 24 h and then transferred in medium with high (20 mM) KH_2_PO_4_ or no iron LIM for 6 h. Cell extracts were subjected to LC-HRMS/MS for untargeted metabolomics. **(B)** Commercially available standards of sugar nucleotides were used to establish optimal performance with low detection limits, allowing fine-tuning of the method. Samples were analyzed using an IMPACII™ high-resolution mass spectrometer (Bruker Daltonics, Bremen, Germany), coupled with a Vanquish UHPLC system (Thermo Scientific). The [M-H]^-^ extracted ion chromatograms were obtained for the following standards: UDP-α-D-galactose/glucose (*m/z* 565.0487), UDP-xylose (*m/z* 535.0379), UDP-N-acetyl-D-glucosamine (*m/z* 606.0749), GDP-mannose (*m/z* 604.0702), and UDP-glucuronic acid (*m/z* 579.0272). Separation of compounds was accomplished by using an InfinityLab Poroshell 120 HILIC-Z column (2.7 µm particle size, 150 x 2.1 mm) from Agilent.

Supplementary Figure 6 The *pho840* deletion mutant is more resistant to high calcium, but susceptible to inhibitors of the calcineurin pathway. The mutants lacking the high affinity phosphate transporters were tested in spot assays for growth in the presence of calcium, the chelator EGTA, and inhibitors of the calcineurin pathway. Concentrations of the added chemicals are indicated. Plates were incubated at 30°C, 37°C or 39°C for 2 d and then transferred into 30°C for another 2 d before being photographed. Assays were repeated three times.

Supplementary Figure 7 The growth of phosphate transporter mutants is influence by calcium levels. **(A)** Growth of the *pho840*Δ*, pho84*Δ*840*Δ and *pho*ΔΔΔ mutants at 48 h in the presence of a range of calcium levels. The assays were performed in 96 well plates and repeated two times with 3 biological replicates each. **(B)** Flow cytometry to measure intracellular calcium in the *pho840*Δ*, pho84*Δ*840*Δ and *pho*ΔΔΔ mutants. All assays were repeated three times.

Supplementary Figure 8 Phosphate influences the transcript levels of *PHO* genes and genes of the UPR. **(A)** Expression of genes for phosphate acquisition. The Log10 fold changes were compared between WT and mutant *pho*ΔΔΔ at different times and conditions (low phosphate, high phosphate_0.5 h and high phosphate_5 h), with WT as a control. **(B)** Transcript levels for the *IRE1*, *HXL1*_1 and *HXL1_2* genes of the UPR. Log10 fold changes were compared between WT and the mutant *pho*ΔΔΔ at different times and conditions (low phosphate, high phosphate_0.5 h and high phosphate_2 h and _5 h), with the low phosphate condition as a control. Three independent experiments were conducted, and the mean and standard deviation are shown. Statistical significance was determined by performing a one-way ANOVA and Tukey’s multiple comparisons in GraphPad Prism 8.0 (GraphPad Software) (ns, non-significant; *p<0.05 **p<0.01 and ***p<0.001).

Supplementary Figure 9 The *pho*ΔΔΔ mutant has altered lipid composition. Heat map of the results of the liquid chromatography and mass spectrometry analysis for the top 50 compounds (n=3), red-to-blue scale represents high to low metabolite levels. The heat map was obtained with MetaboAnalyst 6.0 using log transformation and pareto scaling.

Supplementary Figure 10 Defects in phosphate transporters influence titan cell formation, virulence and fungal burden in infected mice. **(A)** Single and double *pho* mutants were tested for titan cell formation *in vitro*. **(B)** Phagocytosis (after 2 h infection), proliferation (20 h after phagocytosis) and **(C)** replication rate (20 h/0 h) of WT and *pho*ΔΔΔ mutant in the macrophage-like cell line J774.A1. Assays were repeated three times. **(D)** Virulence of the WT strain and two independent *pho*ΔΔΔ triple mutants in a mouse inhalation model of cryptococcosis. BALB/c mice were inoculated with 2 × 10^5^ cells in 50 μl of PBS and sacrificed at 15% weight loss. **(E)** Fungal load in the lungs, liver, spleen, kidney and brains of four mice at the humane point (15% weight loss). *pho*ΔΔΔ*1* and *pho*ΔΔΔ*2* are two independent mutants. Mean and standard deviation are shown. Statistical significance was determined by performing with one-way ANOVA and Tukey’s multiple comparisons in GraphPad Prism 8.0 (GraphPad Software). (ns, non-significant; *p<0.05 **p<0.01 and ***p<0.001).
