# Supplementary material for: Phosphate availability conditions caspofungin tolerance, capsule attachment and titan cell formation in Cryptococcus neoformans
Source: Front Fungal Biol. 2024 Aug 14;5:1447588. doi: 10.3389/ffunb.2024.1447588 (PMC11349702; doi:10.3389/ffunb.2024.1447588)
Supplement: Supplementary file 1 [file DataSheet1.pdf]

Figure S1

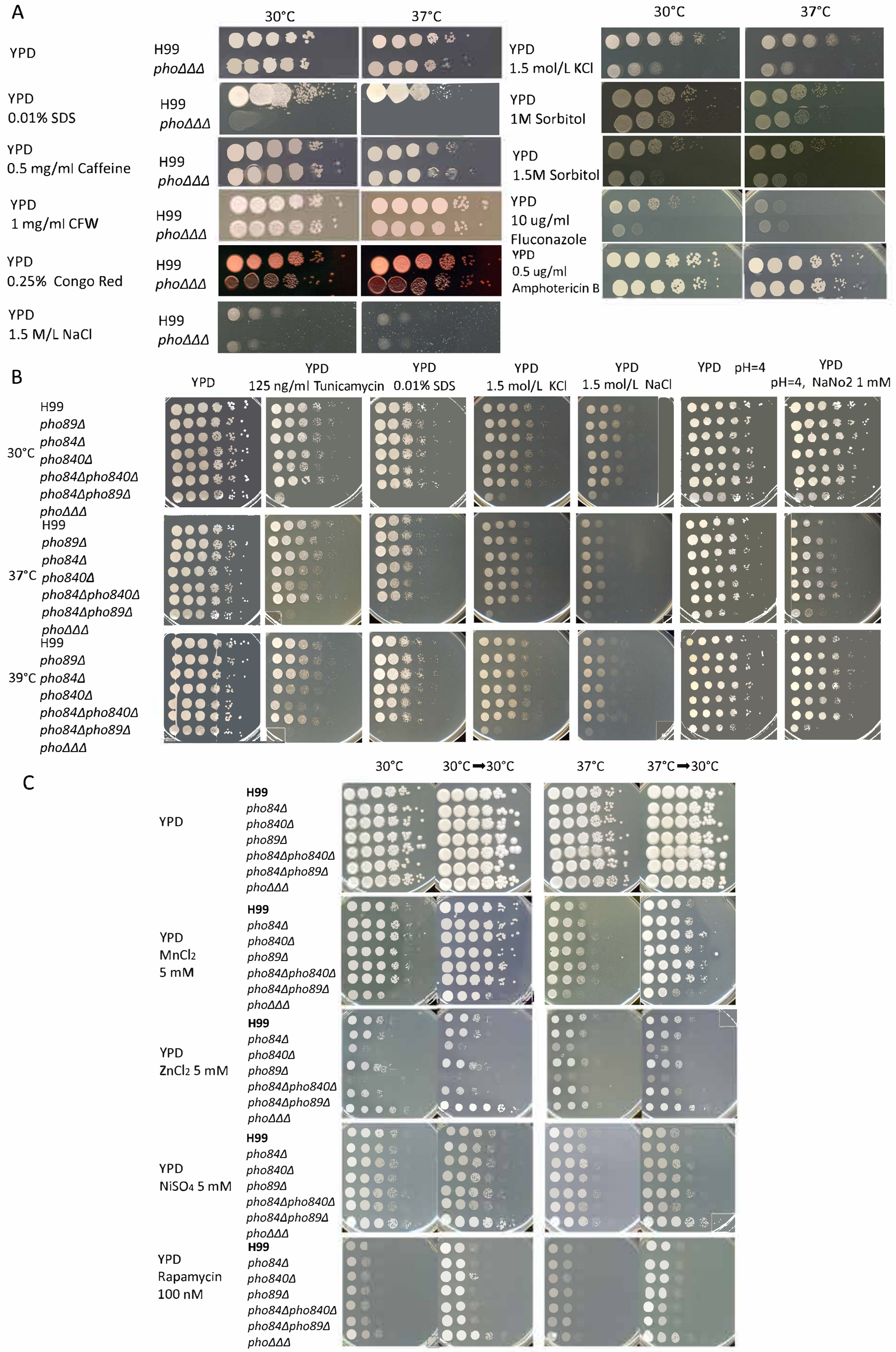
A
30°C
37°C
YPD
H99
phoΔΔΔ
YPD
0.01% SDS
YPD
0.5 mg/ml Caffeine
YPD
1 mg/ml CFW
YPD
0.25% Congo Red
YPD
1.5 M/L NaCl
YPD
1.5 mol/L KCl
YPD
1M Sorbitol
YPD
1.5M Sorbitol
YPD
10 ug/ml
Fluconazole
YPD
0.5 ug/ml
Amphotericin B
B
YPD
YPD
125 ng/ml Tunicamycin
YPD
0.01% SDS
YPD
1.5 mol/L KCl
YPD
1.5 mol/L NaCl
YPD pH=4
YPD
pH=4, NaNo2 1 mM
30°C
37°C
39°C
H99
pho89Δ
pho84Δ
pho840Δ
pho84Δpho840Δ
pho84Δpho89Δ
phoΔΔΔ
C
30°C
30°C➡30°C
37°C
37°C➡30°C
YPD
YPD
MnCl2
5 mM
YPD
ZnCl2 5 mM
YPD
NiSO4 5 mM
YPD
Rapamycin
100 nM
H99
pho84Δ
pho840Δ
pho89Δ
pho84Δpho840Δ
pho84Δpho89Δ
phoΔΔΔ

Figure S2

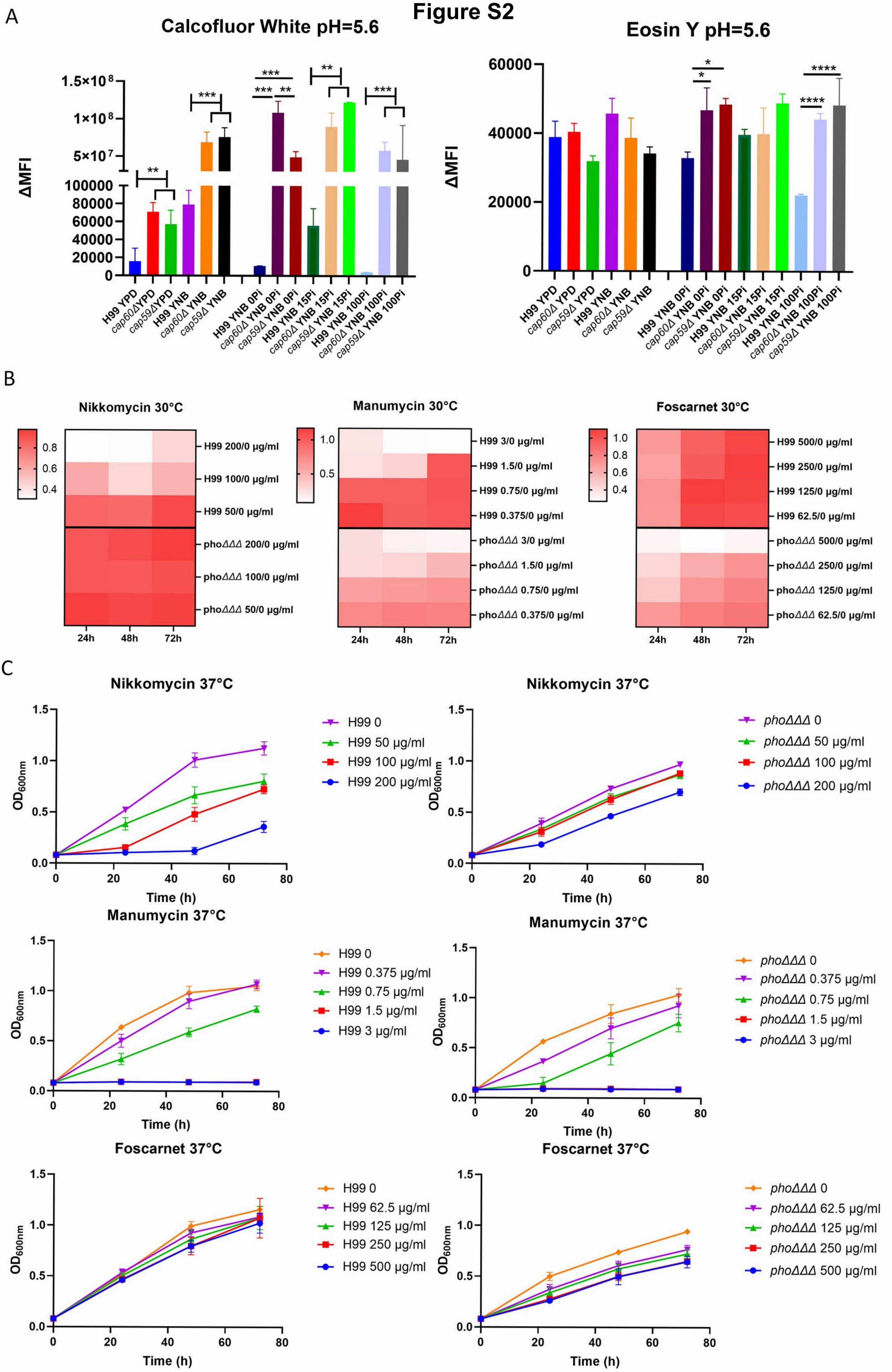

A
Calcofluor White pH=5.6
ΔMFI
H99 YPD
cap60ΔYPD
cap59ΔYPD
H99 YNB
cap60Δ YNB
cap59Δ YNB
H99 YNB 0Pi
cap60Δ YNB 0Pi
cap59Δ YNB 0Pi
H99 YNB 15Pi
cap60Δ YNB 15Pi
cap59Δ YNB 15Pi
H99 YNB 100Pi
cap60Δ YNB 100Pi
cap59Δ YNB 100Pi
Eosin Y pH=5.6
B
Nikkomycin 30°C
H99 200/0 μg/ml
H99 100/0 μg/ml
H99 50/0 μg/ml
phoΔΔΔ 200/0 μg/ml
phoΔΔΔ 100/0 μg/ml
phoΔΔΔ 50/0 μg/ml
Manumycin 30°C
H99 3/0 μg/ml
H99 1.5/0 μg/ml
H99 0.75/0 μg/ml
H99 0.375/0 μg/ml
phoΔΔΔ 3/0 μg/ml
phoΔΔΔ 1.5/0 μg/ml
phoΔΔΔ 0.75/0 μg/ml
phoΔΔΔ 0.375/0 μg/ml
Foscarnet 30°C
H99 500/0 μg/ml
H99 250/0 μg/ml
H99 125/0 μg/ml
H99 62.5/0 μg/ml
phoΔΔΔ 500/0 μg/ml
phoΔΔΔ 250/0 μg/ml
phoΔΔΔ 125/0 μg/ml
phoΔΔΔ 62.5/0 μg/ml
24h
48h
72h
C
Nikkomycin 37°C
OD_{600nm}
Time (h)
H99 0
H99 50 μg/ml
H99 100 μg/ml
H99 200 μg/ml
phoΔΔΔ 0
phoΔΔΔ 50 μg/ml
phoΔΔΔ 100 μg/ml
phoΔΔΔ 200 μg/ml
Manumycin 37°C
H99 0
H99 0.375 μg/ml
H99 0.75 μg/ml
H99 1.5 μg/ml
H99 3 μg/ml
phoΔΔΔ 0
phoΔΔΔ 0.375 μg/ml
phoΔΔΔ 0.75 μg/ml
phoΔΔΔ 1.5 μg/ml
phoΔΔΔ 3 μg/ml
Foscarnet 37°C
H99 0
H99 62.5 μg/ml
H99 125 μg/ml
H99 250 μg/ml
H99 500 μg/ml
phoΔΔΔ 0
phoΔΔΔ 62.5 μg/ml
phoΔΔΔ 125 μg/ml
phoΔΔΔ 250 μg/ml
phoΔΔΔ 500 μg/ml

Figure S3

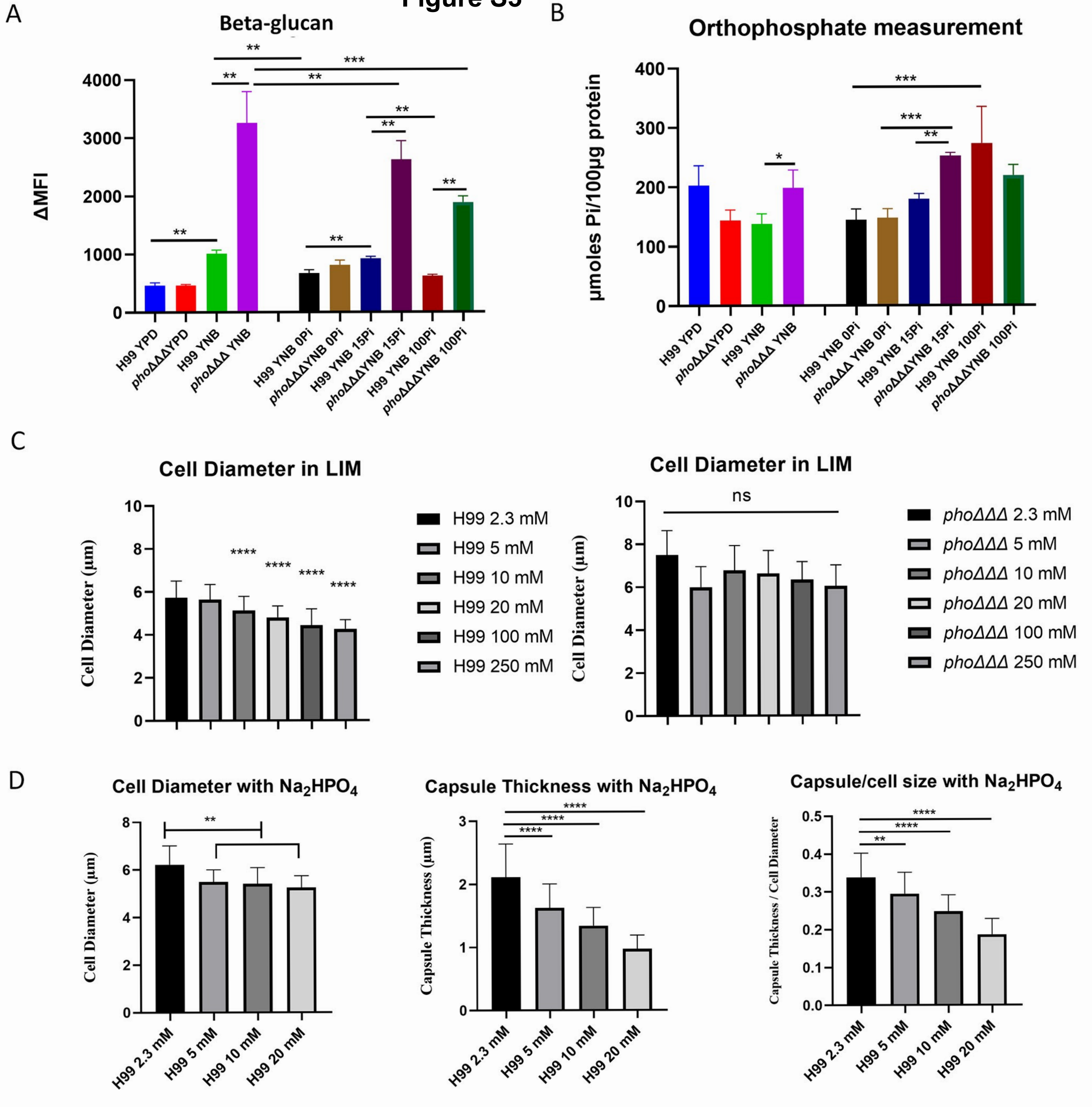
A
Beta-glucan
ΔMFI
H99 YPD
phoΔΔΔYPD
H99 YNB
phoΔΔΔ YNB
H99 YNB 0Pi
phoΔΔΔYNB 0Pi
H99 YNB 15Pi
phoΔΔΔYNB 15Pi
H99 YNB 100Pi
phoΔΔΔYNB 100Pi
B
Orthophosphate measurement
μmoles Pi/100μg protein
H99 YPD
phoΔΔΔYPD
H99 YNB
phoΔΔΔ YNB
H99 YNB 0Pi
phoΔΔΔ YNB 0Pi
H99 YNB 15Pi
phoΔΔΔYNB 15Pi
H99 YNB 100Pi
phoΔΔΔYNB 100Pi
C
Cell Diameter in LIM
Cell Diameter (μm)
H99 2.3 mM
H99 5 mM
H99 10 mM
H99 20 mM
H99 100 mM
H99 250 mM
Cell Diameter in LIM
ns
phoΔΔΔ 2.3 mM
phoΔΔΔ 5 mM
phoΔΔΔ 10 mM
phoΔΔΔ 20 mM
phoΔΔΔ 100 mM
phoΔΔΔ 250 mM
D
Cell Diameter with Na2HPO4
Cell Diameter (μm)
Capsule Thickness with Na2HPO4
Capsule Thickness (μm)
Capsule/cell size with Na2HPO4
Capsule Thickness / Cell Diameter
H99 2.3 mM
H99 5 mM
H99 10 mM
H99 20 mM

Figure S4

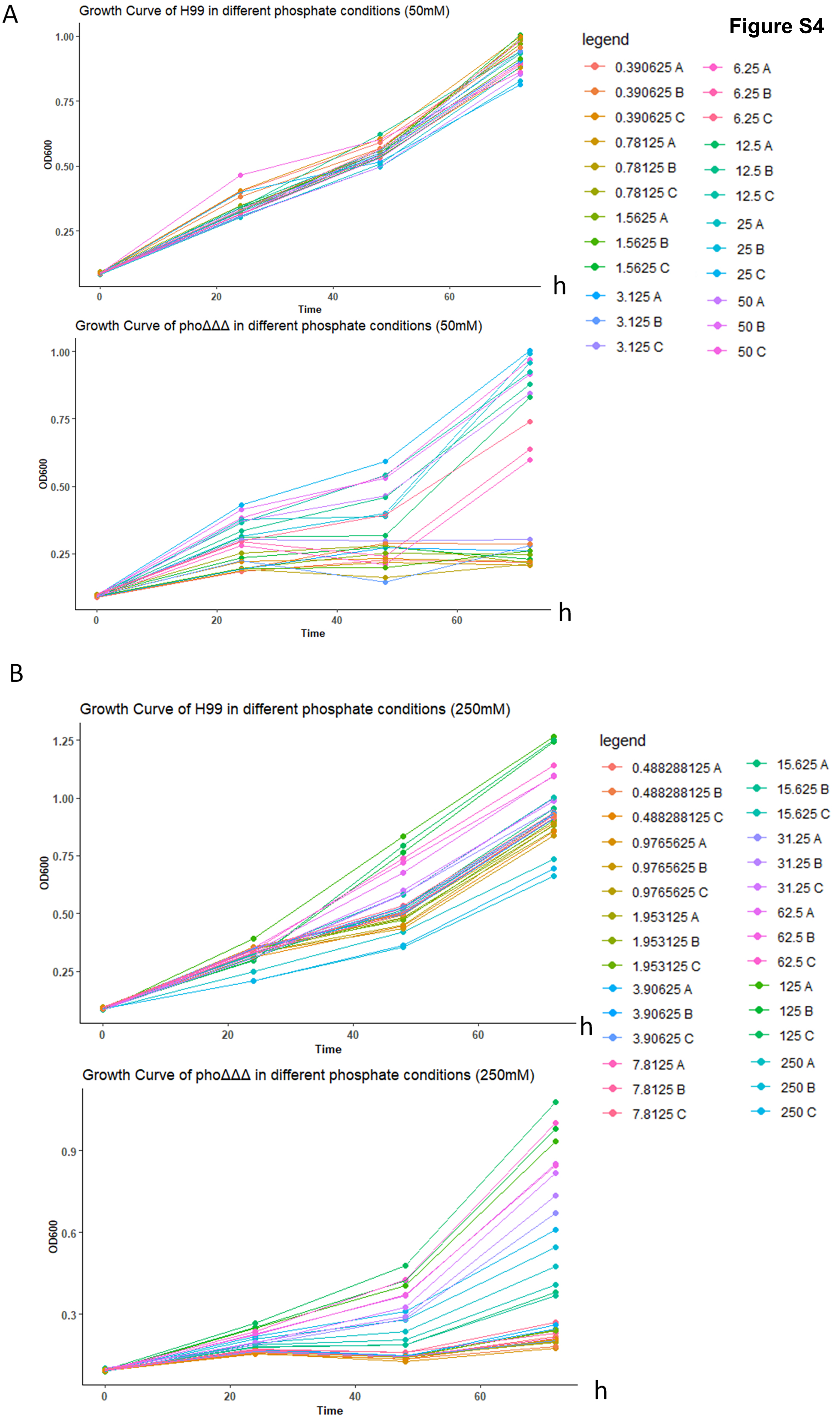
A
Growth Curve of H99 in different phosphate conditions (50mM)
OD600
Time
h
legend
0.390625 A
0.390625 B
0.390625 C
0.78125 A
0.78125 B
0.78125 C
1.5625 A
1.5625 B
1.5625 C
3.125 A
3.125 B
3.125 C
6.25 A
6.25 B
6.25 C
12.5 A
12.5 B
12.5 C
25 A
25 B
25 C
50 A
50 B
50 C
Growth Curve of phoΔΔΔ in different phosphate conditions (50mM)
OD600
Time
h
B
Growth Curve of H99 in different phosphate conditions (250mM)
OD600
Time
h
Growth Curve of phoΔΔΔ in different phosphate conditions (250mM)
OD600
Time
h
legend
0.488288125 A
0.488288125 B
0.488288125 C
0.9765625 A
0.9765625 B
0.9765625 C
1.953125 A
1.953125 B
1.953125 C
3.90625 A
3.90625 B
3.90625 C
7.8125 A
7.8125 B
7.8125 C
15.625 A
15.625 B
15.625 C
31.25 A
31.25 B
31.25 C
62.5 A
62.5 B
62.5 C
125 A
125 B
125 C
250 A
250 B
250 C

A

Figure S5

Top 20 pathways
Alanine, aspartate and glutamate metabolism
Arginine biosynthesis
Amino sugar and nucleotide sugar metabolism
Galactose metabolism
Citrate cycle (TCA cycle)
Lysine biosynthesis
Biosynthesis of various plant secondary metabolites
Glyoxylate and dicarboxylate metabolism
Purine metabolism
Cyanoamino acid metabolism
Nicotinate and nicotinamide metabolism
Valine, leucine and isoleucine biosynthesis
Cysteine and methionine metabolism
Fructose and mannose metabolism
Starch and sucrose metabolism
Arginine and proline metabolism
Pyrimidine metabolism
Glutathione metabolism
Lysine degradation
Valine, leucine and isoleucine degradation

B **standards**

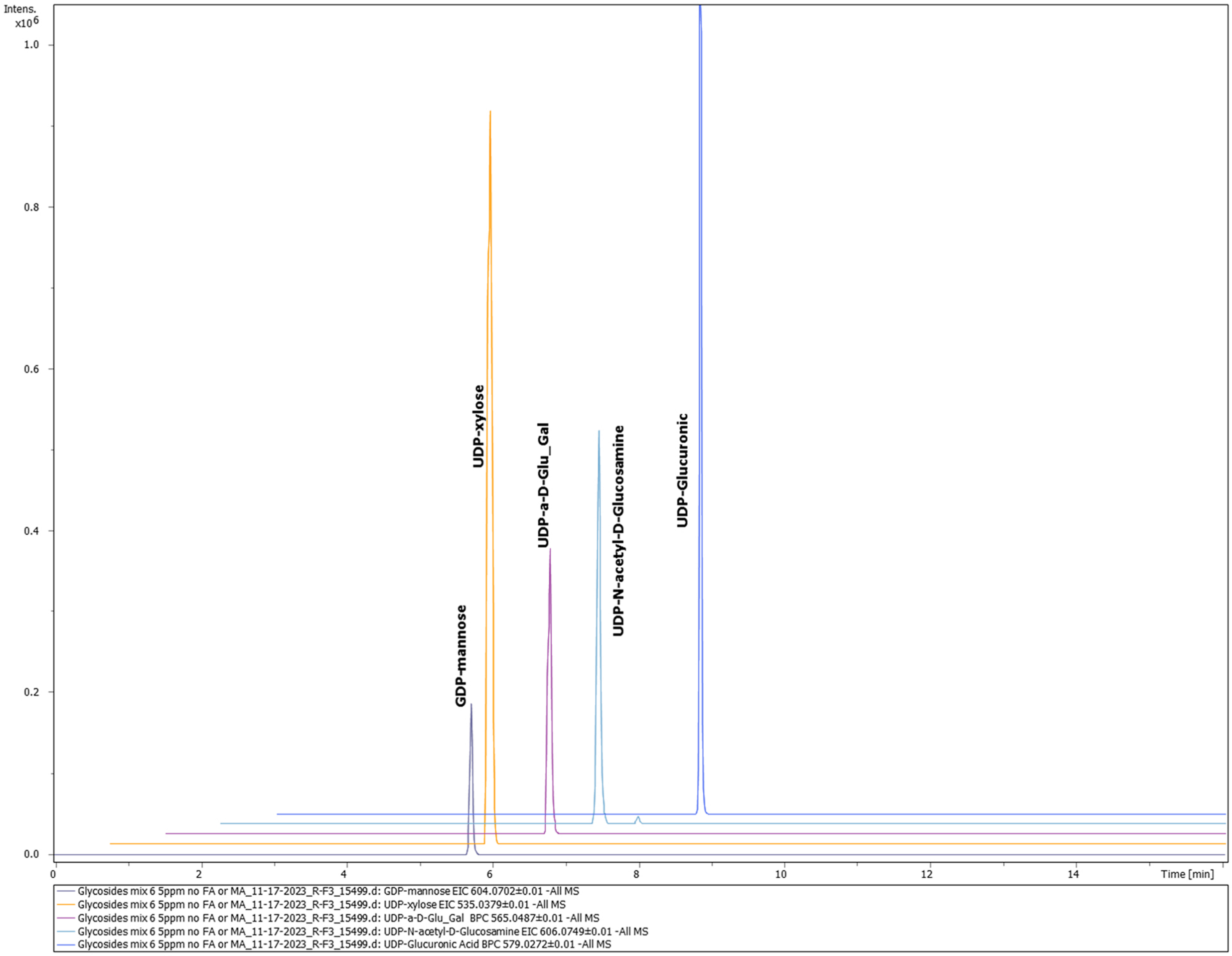

Figure S6

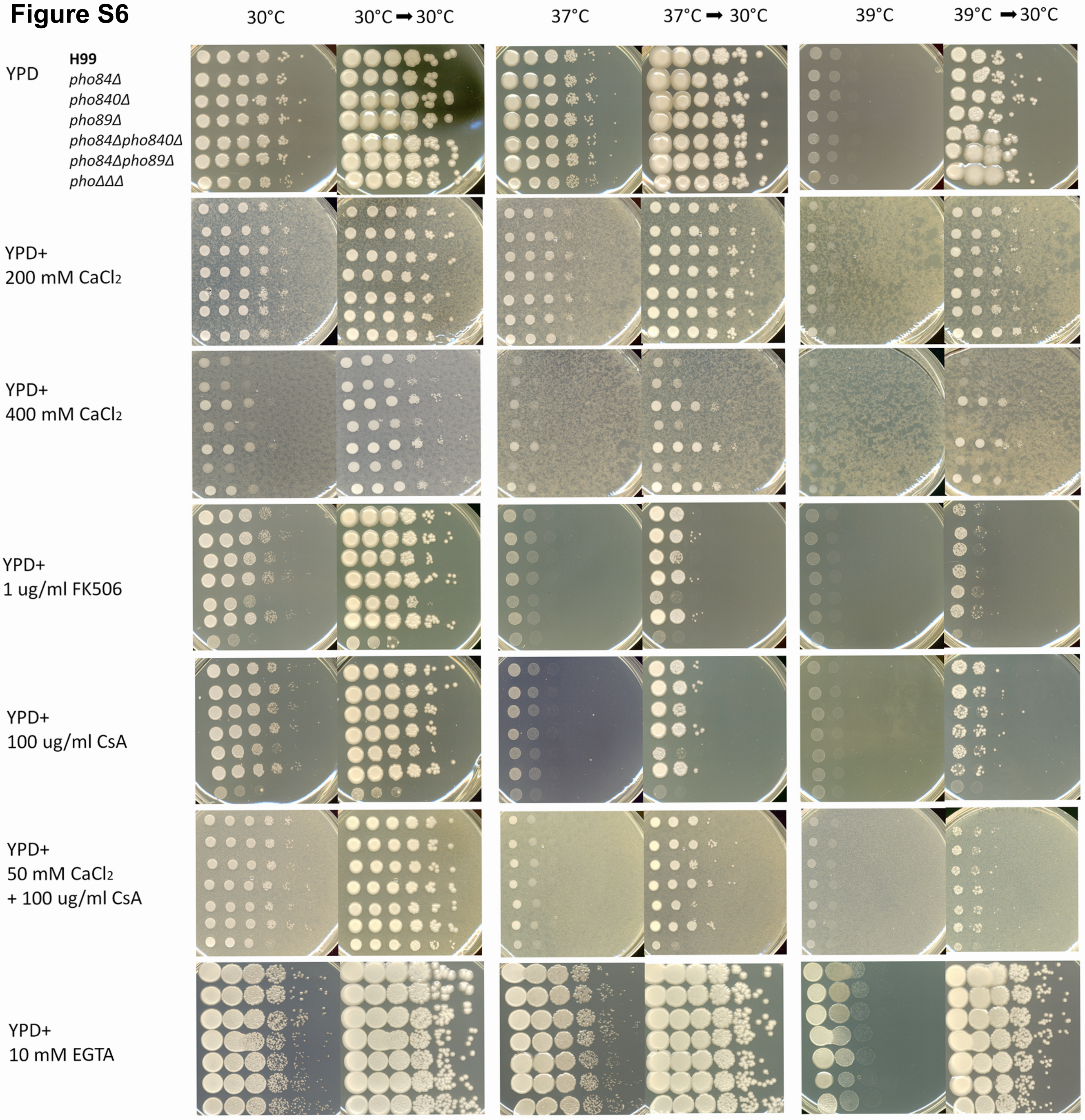
30°C
30°C ➡ 30°C
37°C
37°C ➡ 30°C
39°C
39°C ➡ 30°C
YPD
H99
pho84Δ
pho840Δ
pho89Δ
pho84Δpho840Δ
pho84Δpho89Δ
phoΔΔΔ
YPD+
200 mM CaCl2
YPD+
400 mM CaCl2
YPD+
1 ug/ml FK506
YPD+
100 ug/ml CsA
YPD+
50 mM CaCl2
+ 100 ug/ml CsA
YPD+
10 mM EGTA

Figure S7

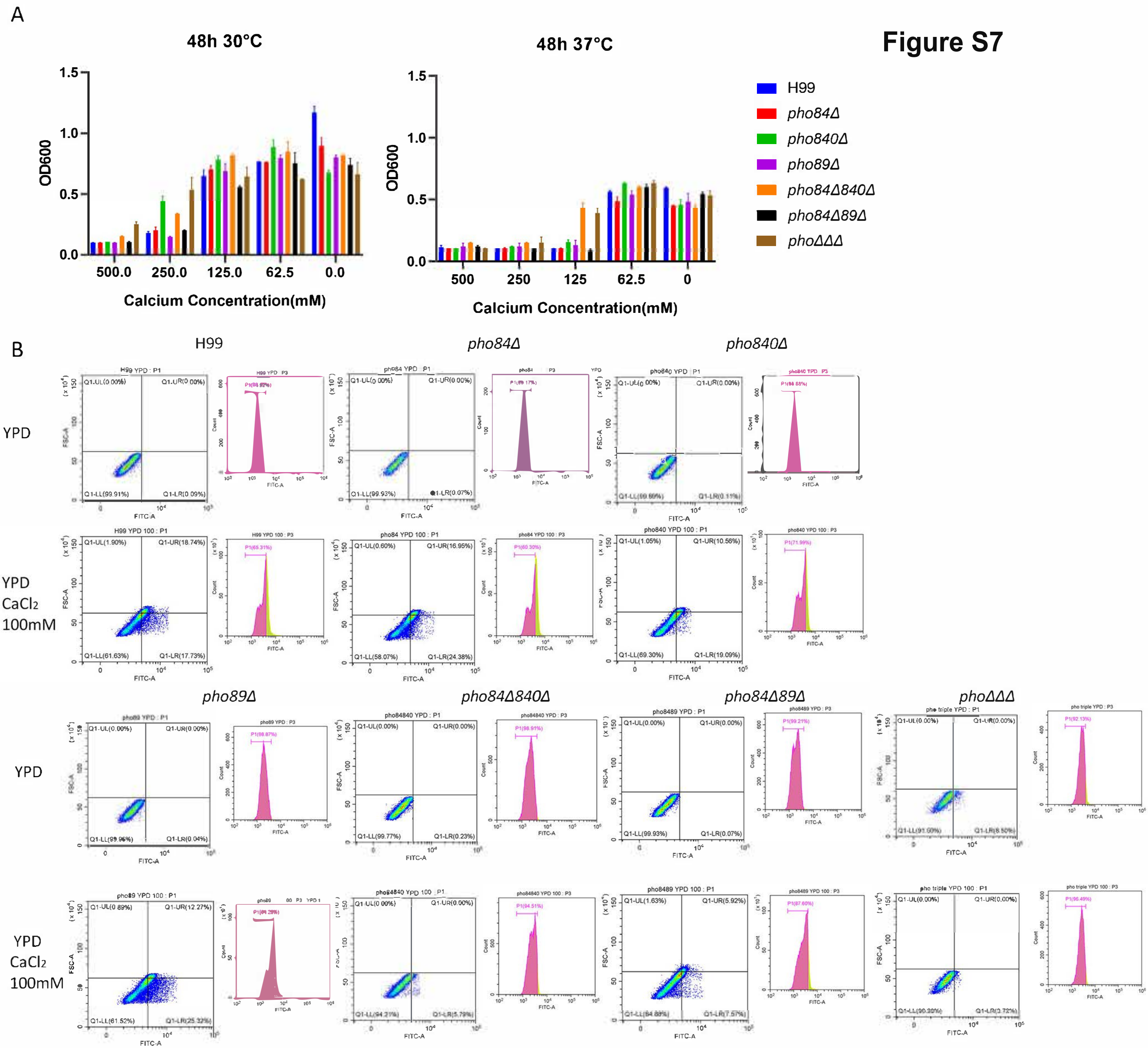
A
48h 30°C
48h 37°C
OD600
1.5
1.0
0.5
0.0
500.0
250.0
125.0
62.5
0.0
500
250
125
62.5
0
Calcium Concentration(mM)
H99
pho84Δ
pho840Δ
pho89Δ
pho84Δ840Δ
pho84Δ89Δ
phoΔΔΔ
B
H99
pho84Δ
pho840Δ
YPD
YPD CaCl2 100mM
pho89Δ
pho84Δ840Δ
pho84Δ89Δ
phoΔΔΔ
YPD
YPD CaCl2 100mM
FITC-A
FSC-A
Count

Figure S8

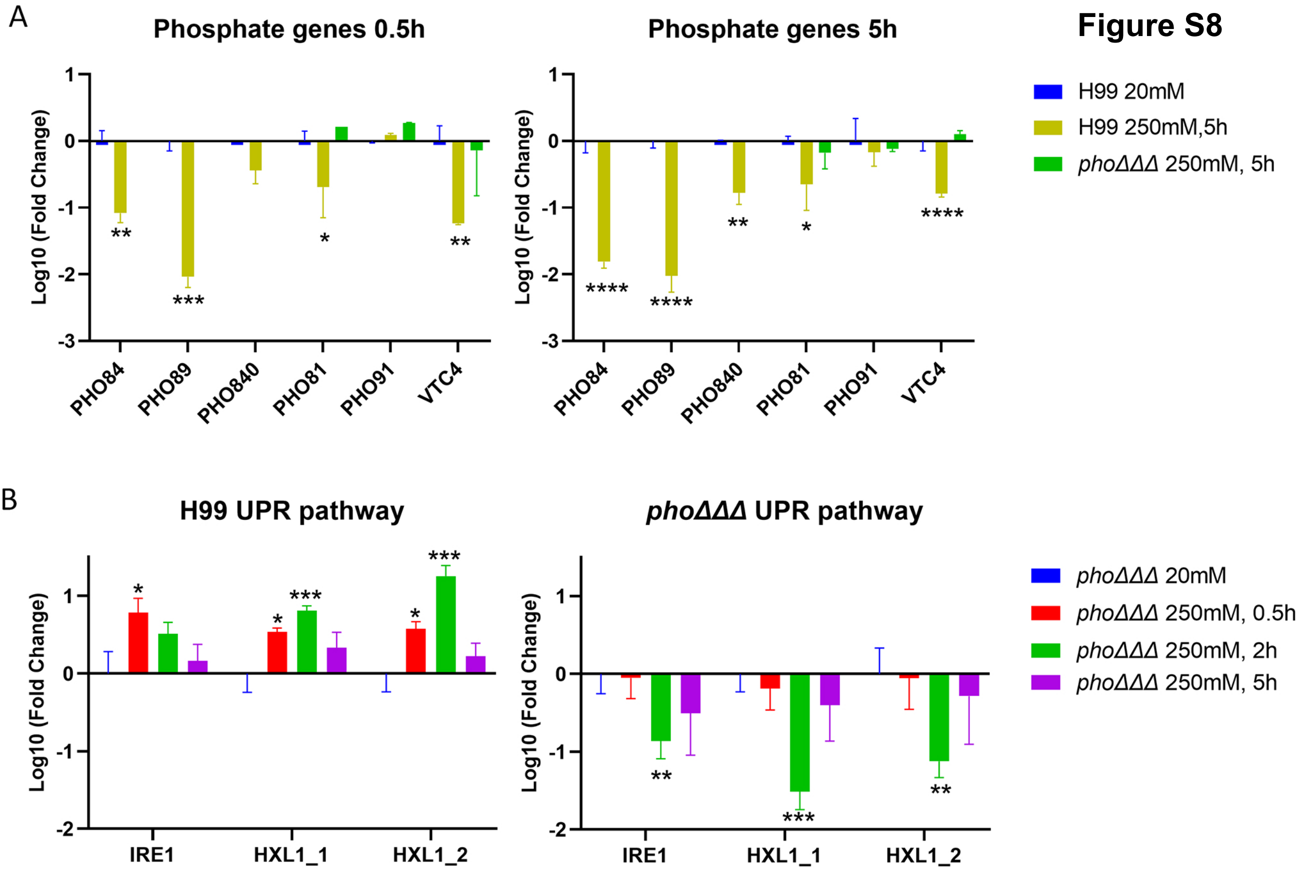
A
Phosphate genes 0.5h
Phosphate genes 5h
Log10 (Fold Change)
PHO84
PHO89
PHO840
PHO81
PHO91
VTC4
H99 20mM
H99 250mM,5h
phoΔΔΔ 250mM, 5h
B
H99 UPR pathway
phoΔΔΔ UPR pathway
IRE1
HXL1_1
HXL1_2
phoΔΔΔ 20mM
phoΔΔΔ 250mM, 0.5h
phoΔΔΔ 250mM, 2h
phoΔΔΔ 250mM, 5h

Figure S9

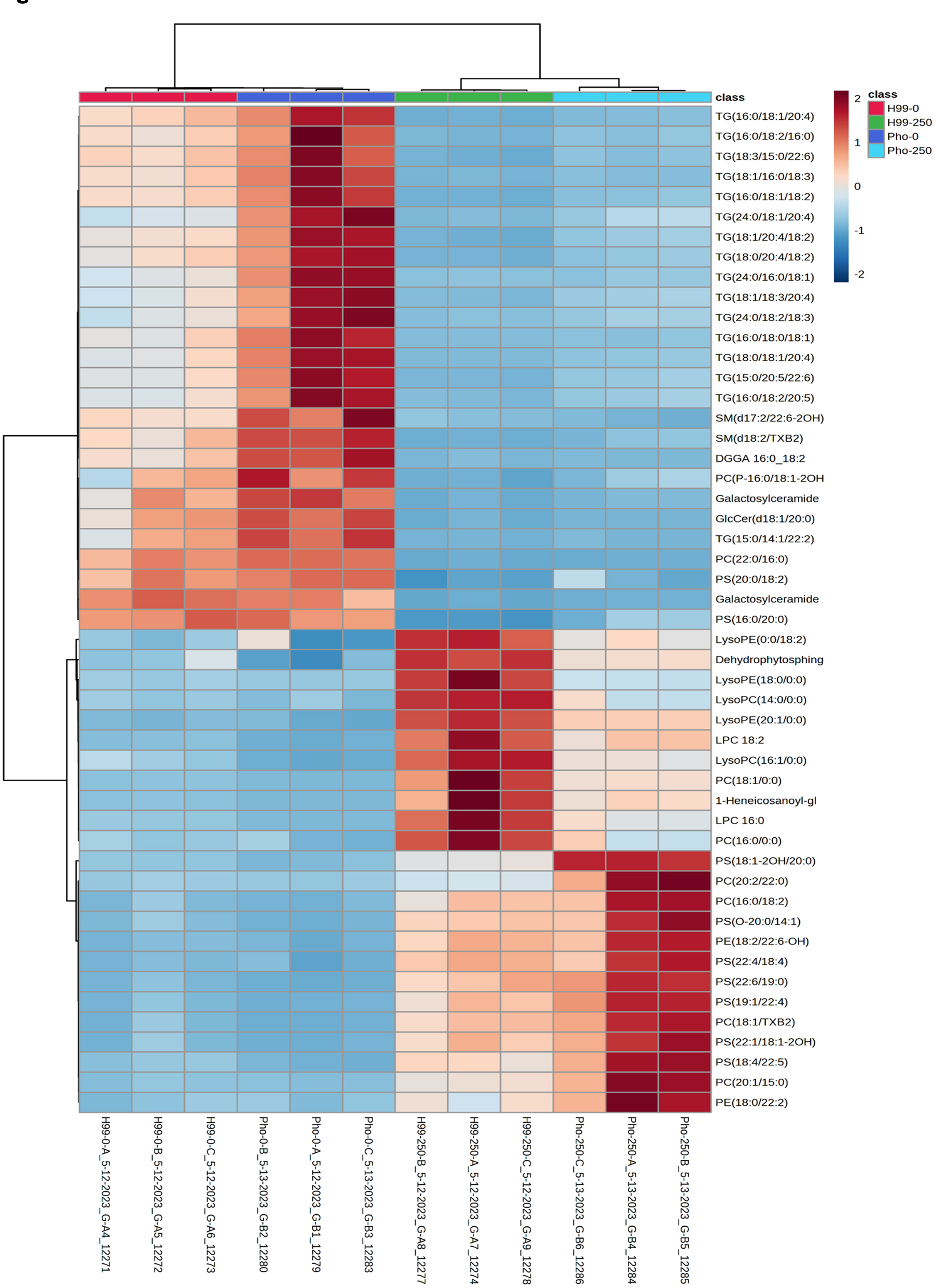
TOP 50
class
2
1
0
-1
-2
class
H99-0
H99-250
Pho-0
Pho-250
TG(16:0/18:1/20:4)
TG(16:0/18:2/16:0)
TG(18:3/15:0/22:6)
TG(18:1/16:0/18:3)
TG(16:0/18:1/18:2)
TG(24:0/18:1/20:4)
TG(18:1/20:4/18:2)
TG(18:0/20:4/18:2)
TG(24:0/16:0/18:1)
TG(18:1/18:3/20:4)
TG(24:0/18:2/18:3)
TG(16:0/18:0/18:1)
TG(18:0/18:1/20:4)
TG(15:0/20:5/22:6)
TG(16:0/18:2/20:5)
SM(d17:2/22:6-2OH)
SM(d18:2/TXB2)
DGGA 16:0_18:2
PC(P-16:0/18:1-2OH
Galactosylceramide
GlcCer(d18:1/20:0)
TG(15:0/14:1/22:2)
PC(22:0/16:0)
PS(20:0/18:2)
Galactosylceramide
PS(16:0/20:0)
LysoPE(0:0/18:2)
Dehydrophytosphing
LysoPE(18:0/0:0)
LysoPC(14:0/0:0)
LysoPE(20:1/0:0)
LPC 18:2
LysoPC(16:1/0:0)
PC(18:1/0:0)
1-Heneicosanoyl-gl
LPC 16:0
PC(16:0/0:0)
PS(18:1-2OH/20:0)
PC(20:2/22:0)
PC(16:0/18:2)
PS(O-20:0/14:1)
PE(18:2/22:6-OH)
PS(22:4/18:4)
PS(22:6/19:0)
PS(19:1/22:4)
PC(18:1/TXB2)
PS(22:1/18:1-2OH)
PS(18:4/22:5)
PC(20:1/15:0)
PE(18:0/22:2)
H99-0-A_5-12-2023_G-A4_12271
H99-0-B_5-12-2023_G-A5_12272
H99-0-C_5-12-2023_G-A6_12273
Pho-0-B_5-13-2023_G-B2_12280
Pho-0-A_5-12-2023_G-B1_12279
Pho-0-C_5-13-2023_G-B3_12283
H99-250-B_5-12-2023_G-A8_12277
H99-250-A_5-12-2023_G-A7_12274
H99-250-C_5-12-2023_G-A9_12278
Pho-250-C_5-13-2023_G-B6_12286
Pho-250-A_5-13-2023_G-B4_12284
Pho-250-B_5-13-2023_G-B5_12285

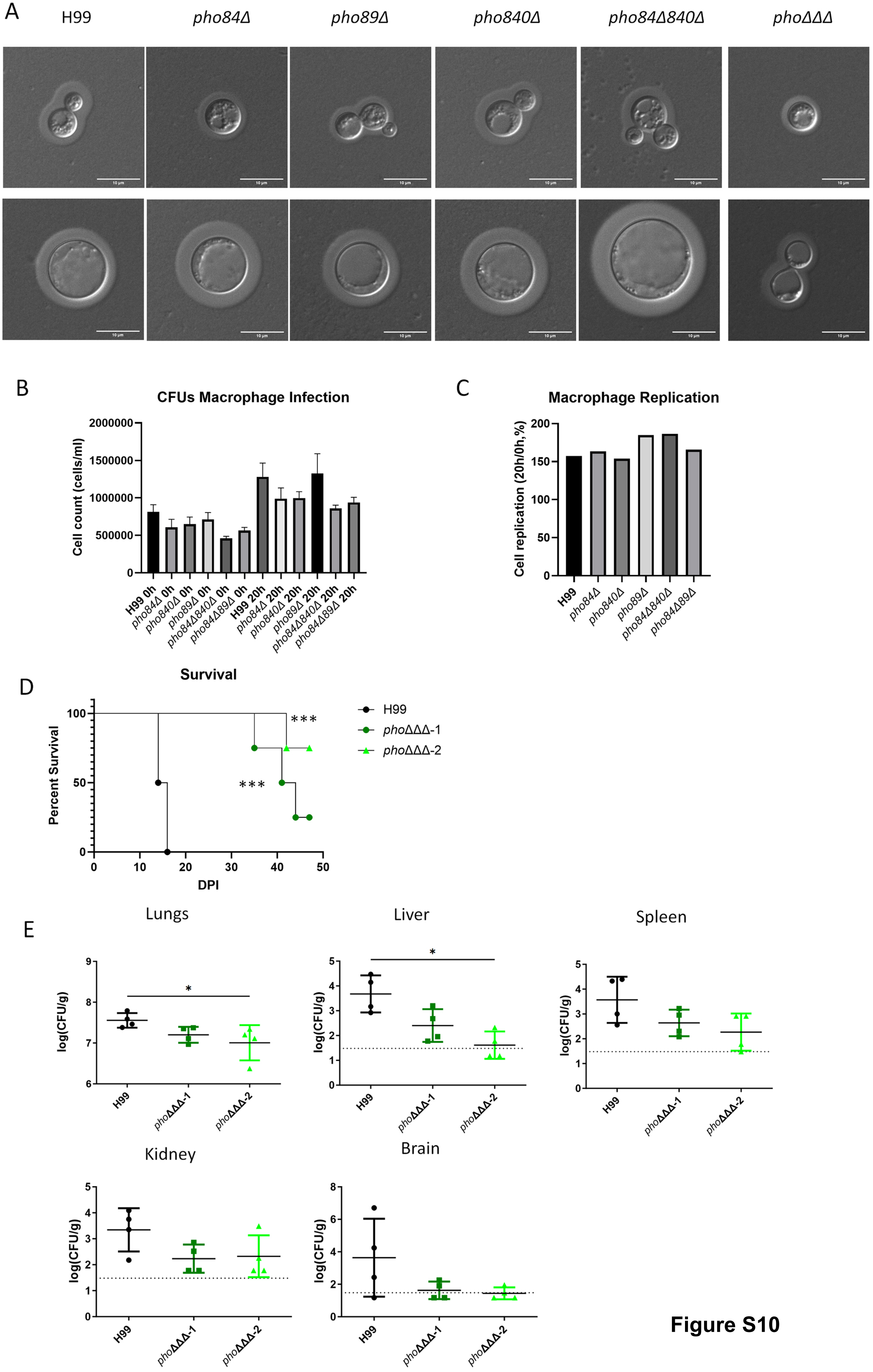
A
H99
pho84Δ
pho89Δ
pho840Δ
pho84Δ840Δ
phoΔΔΔ
B
CFUs Macrophage Infection
Cell count (cells/ml)
H99 0h
pho84Δ 0h
pho840Δ 0h
pho89Δ 0h
pho84Δ840Δ 0h
pho84Δ89Δ 0h
H99 20h
pho84Δ 20h
pho840Δ 20h
pho89Δ 20h
pho84Δ840Δ 20h
pho84Δ89Δ 20h
C
Macrophage Replication
Cell replication (20h/0h,%)
D
Survival
Percent Survival
DPI
H99
phoΔΔΔ-1
phoΔΔΔ-2
E
Lungs
Liver
Spleen
Kidney
Brain
log(CFU/g)

Figure S10

Supplemental Table S1. Primers used for qPCR.

Gene	Primers	Sequence (5' to 3')
CIR1	cir1_F	GATAAGTCGGAGGCTGAACG
	cir1_R	TCTCCGTCCCACTGACTACC
PKA1	pka1_F	TCCGCTATCCGACATACTTTG
	pka1_R	GAATTTCGCGCCTGTACAAC
PKR1	pkr1_F	CCTAAATCGCAGGACACGAG
	pkr1_R	CCCTGTCGAAAAACCGTAAC
ACT1	act1_F	AAGGAGATCACCGCCCTTG
	act1_R	GGGACCAGACTCGTCGTATTC
GAPDH	gapdh_F	GGACCTCGTTTGTCGCATTG
	gapdh_R	AGCCTGGGCATCGAAGATAG
PHO84	pho84_F	CCTACTCGTTACCGATCAACTG
	pho84_R	AGTCTCGGGAAGAAGCAATG
VTC4	vtc4_F	GATGCCGTCGGTATGGTTTC
	vtc4_R	TAACAACGCCGCGCAAAG
PHO840	pho840_F	CCTTCCCGCCGTTATCTAC
	pho840_R	GATACTCGTGTGCCCTACC
PHO89	pho89_F	GTGCTCGGTAACAGACTGAC
	pho89_R	ACGCTCGCCAGTTAATCG
IRE1	ire1_F	TGCAGAAGATGGCGTTGC
	ire1_R	ACACTCCCGCCTTTATAC
HXL1_1	Hxl1_F_1	ATGGCTACCGCTGTCGCT
	Hxl1_R_1	TGATTCGCGGTTACGGAT
HXL1_2	Hxl1_F_2	CACTCCATTCCTTTCTGC
	Hxl1_R _2	CGTAACTCCACTGTGTCC

Supplemental Table S2. Conditions for flow cytometry and microscopy

Flow cytometry	O/N	Treatment media	Washing	Staining Dye	Staining time	Washing	Flow channel
**Membrane permeability**	**YPD**	**YPD with 0.005% or 0.01% SDS, 3h room temperature**	**Twice with PBS**	**5ug/ml Propidium Iodide**	**dark, 37°C, 30 min**	**Three with PBS**	**phycoerythrin (PE), (493/636 nm)**
**Intracellular calcium detection**	**YPD**	**YPD with 100mM $CaCl_2$, 2h in room temperature**	**Twice with PBS**	**5 µM calcium indicator Cal Green 1 AM**	**dark, 37°C, 30 min**	**Three times with PBS**	**Fluorescein isothiocyan-ate (FITC, 488/530 nm)**
**Chitin Detection**	**YPD**	**YNB media (2% glucose, various Pi), 24h at 30°C**	**Twice with McIlvaine's buffer[1]**	**100 µg/mL of CFW (calcofluor white)**	**dark, 30°C, 10 min**	**Three times with dH_2O**	**Pacific Blue (PB450, 405/450 nm)**
**Chitosan Detection**	**YPD**	**YNB media (2% glucose, various Pi), 24h at 30°C**	**Twice with McIlvaine's buffer**	**250 ug/ml Eosin Y**	**dark, room temperature, 15 min**	**Three times with dH_2O**	**Fluorescein isothiocyan-ate (FITC, 488/530 nm)**
**Beta-glucan Detection**	**YPD**	**YNB media (2% glucose, various Pi), 24h at 30°C**	**Twice with dH_2O**	**0.05% Aniline blue**	**dark, room temperature, 10 min**	**Three times with dH_2O**	**Pacific Blue (PB450, 405/450 nm)**
**Detection of Titan cells**	**YPD**	**MM (800rpm, 30°C) for 72h**	**MM**	**no**	**no**	**Three times with dH_2O**	**FSC/SSC high for titan cell, FSC/SSC low for normal cells (tC)**
**Microscopy**	**overnight**	**Treatment media**	**Washing**	**Staining Dye**	**Staining time**	**Washing**	**Microscope channel**
**Chitin**	**YPD**	**MM for titan cell, YPD for normal cell**	**Twice with PBS**	**0.0001 µg/mL CFW in PBS**	**dark, room temperature, 10 min**	**Three times with PBS**	**Blue**
**Capsule**	**YPD**	**MM for titan cell, YPD for normal cell**	**Twice with PBS**	**10ug/ml 18B7 monoclon-al antibody plus 1:500 Alexa flour 568 antibody**	**blocked 1 hour at 30°C. [2]**	**Three times with block-ing buffer**	**Alexa fluor 568**

[1]McIlvaine's buffer and concentrations were employed based on previous studies: Santiago-Tirado, F., Peng, T., Yang, M., Hang, H., and Doering, T. (2015). A single protein S-acyl transferase acts through diverse substrates to determine Cryptococcal morphology, stress tolerance, and pathogenic outcome. PLoS Pathog, 11(5), e1004908. doi:10.1371/journal.ppat.1004908.

Baker, L., Specht, C., Donlin, M., and Lodge, J. (2007). Chitosan, the deacetylated form of chitin, is necessary for cell wall integrity in *Cryptococcus neoformans*. Eukaryot. Cell., 6(5), 855-67. doi:10.1128/EC.00399-06

Washing with dH_20 after starvation in no phosphate was used to avoid the effects of phosphate from McIlvaine's buffer.

[2]Cells were blocked for 1 hour at 30°C (0.5% albumin in 1 x TBS), incubated with 10 µg/ml 18B7 monoclonal antibody at 30°C for 1 h, washed with blocking buffer twice, and incubated for 1 h with 1:500 with Alexa flour 568 antibody (ThermoFisher) at room temperature in the dark.
